# Bone morphogenetic proteins, breast cancer, and bone metastases: striking the right balance

**DOI:** 10.1530/ERC-17-0139

**Published:** 2017-07-21

**Authors:** Catherine Zabkiewicz, Jeyna Resaul, Rachel Hargest, Wen Guo Jiang, Lin Ye

**Affiliations:** Cardiff China Medical Research CollaborativeCardiff University School of Medicine, Cardiff, UK

**Keywords:** bone morphogenetic protein, breast cancer, bone metastasis and tumour biology

## Abstract

Bone morphogenetic proteins (BMPs) belong to the TGF-β super family, and are essential for the regulation of foetal development, tissue differentiation and homeostasis and a multitude of cellular functions. Naturally, this has led to the exploration of aberrance in this highly regulated system as a key factor in tumourigenesis. Originally identified for their role in osteogenesis and bone turnover, attention has been turned to the potential role of BMPs in tumour metastases to, and progression within, the bone niche. This is particularly pertinent to breast cancer, which commonly metastasises to bone, and in which studies have revealed aberrations of both BMP expression and signalling, which correlate clinically with breast cancer progression. Ultimately a BMP profile could provide new prognostic disease markers. As the evidence suggests a role for BMPs in regulating breast tumour cellular function, in particular interactions with tumour stroma and the bone metastatic microenvironment, there may be novel therapeutic potential in targeting BMP signalling in breast cancer. This review provides an update on the current knowledge of BMP abnormalities and their implication in the development and progression of breast cancer, particularly in the disease-specific bone metastasis.

## Introduction

Breast cancer is the most common cancer in women worldwide (Ferlay *et al*. 2015). In developed countries, it receives media coverage and research funding above all other cancers (Konfortion *et al*. 2014, American Cancer Society 2016) and thus continual progress is made in understanding tumour biology, developing diagnostics and improved therapeutics. Despite progress, 15% of patients diagnosed with metastatic breast cancer survive 5 years, compared to 99% of stage I breast cancers (Cancer Research UK 2017). Even for those treated at an early stage, there is still a significant risk of relapse, often several years later. This is particularly true of oestrogen receptor-positive breast cancers, which are at a particular risk of late relapse (Yamashita *et al*. 2016). The leading metastatic site is bone, which holds the majority of tumour burden at death (Awolaran *et al*. 2016). Osteolytic lesions lead to bone pain, fractures, spinal cord compression and hypercalcaemia, reducing quality and length of life for the patient. Symptomatic management includes inhibition of osteoclast activity, swinging the balance of bone turnover away from osteolysis.

Known since 1965 as a key regulator of bone development and turnover, bone morphogenetic proteins (BMPs) have more recently been implicated in bone metastasis of many solid tumours (Ye *et al*. 2007*b*, Davis *et al*. 2016). Given the propensity for breast cancer to metastasise to bone makes BMPs of particular interest in this area. Their influence on breast tumour biology may also extend well beyond the bone microenvironment, opening avenues for targeted therapies that could reduce metastatic potential. Here, we review the current knowledge regarding BMPs role in breast cancer progression, metastasis and relapse.

## BMP signalling aberrations in breast cancer

### BMP signalling pathway

**Figure 1:**
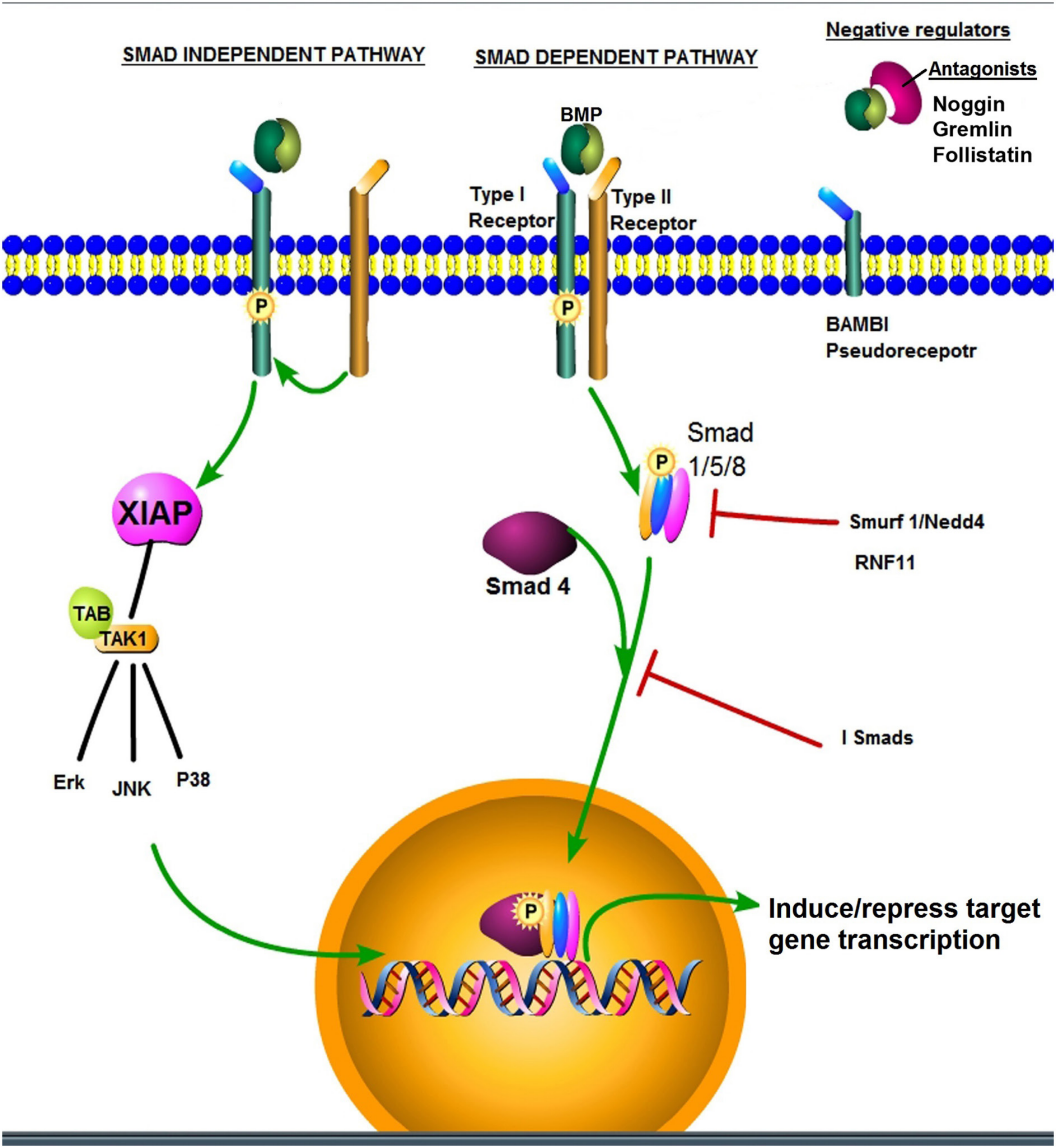
BMP signalling utilises canonical Smad dependant pathways and non-canonical Smad-independent pathways. According to combination and sequence of receptor complex formation or recruitment if the BMP ligand (two tone sphere) binds to a preformed heteromeric receptor complex this results in Smad dependant signalling. The Smad 1/5/8 complex binds to Smad 4 and translocates to the nucleus whereby they interact with transcription factors, or directly binding to DNA to regulate gene transcription. If the BMP binds to a type I receptor and recruits the type 2 receptor to the complex, Smad-independent signalling results. There are multiple levels of regulation, including inhibitory Smads 6 and 7 (I-Smads), pseudoreceptors lacking the serine/threonine kinase domain (BAMBI), ubiquitination and degradation (Smurf/Nedd4/RNF11) and target gene transcription of negative regulators (such as the BMP antagonists) functioning as negative feedback. There are myriad other BMP target genes in breast cancer such as ID1, Snail, Zeb1, p21, PTEN, MMPs and ER that affect cancer cell functions. The figure was made with pathway builder tools from www.proteinlounge.com. A full colour version of the figure is available at http://dx.doi.org/10.1530/ERC-17-0139

BMPs are members of the TGF-β super family, which regulate cellular differentiation, proliferation, apoptosis and motility, particularly in embryonic development and tissue homeostasis (Nohe *et al*. 2004, Ye *et al*. 2009, Davis *et al*. 2016). Binding to a complex of serine-threonine kinase transmembrane receptors comprising Type I and Type II receptors induces intracellular signalling through the pathway-restricted Smads (R-Smads-Smads 1, 5 and 8) and Smad-4, which assists the translocation into nucleus, thus regulating BMP responsive genes in association with transcriptional co-activators or co-repressors. This pathway is known as the Smad dependent or canonical pathway. In noncanonical BMP signalling, the receptor complex is instead recruited as a result of ligand binding, triggering a Smad-independent pathway, which involves various branches of the mitogen-activated protein kinase (MAPK) pathway, RAS pathways, PI3K/Akt pathways, P/KC pathways and Rho-GTPases pathways, dependant on both the BMP ligand and receptors recruited (Derynck & Feng 1997, Nohe *et al*. 2004, Ye *et al*. 2009, Bragdon *et al*. 2011, Davis *et al*. 2016). As a vital embryonic pathway, several layers of inhibition and control are important for normal tissue development and add further to the great plasticity of BMP signalling (Fig. 1).

### Regulation of BMP signalling

BAMBI (BMP and activin membrane bound inhibitor) is a pseudoreceptor related to type I receptors, which limits BMP function. It is present in breast cancer cell lines and expression has been noted as upregulated in cancers, but as it also abrogates TGF-β signalling, the pro-oncogenic effect may not be specific to BMPs (Wang 2015). I-Smads (inhibitory Smads) such as Smad 6 and 7 prevent complex formation between R-Smads and Smad-4, thus affecting transcriptional regulation in the nucleus, which was well reviewed previously (Miyazono 2008, Bragdon *et al*. 2011). They bind to activated type I receptors blocking activity and promoting the degradation of receptors through ubiquitination pathways (Nohe *et al*. 2004).

Smad ubiquitination regulatory factor 1 (Smurf1) is the ligase of Smad 1/5/8, amongst other substrates. In turn, Smurf2 induces degradation of Smurf 1. Downregulation of Smurf1 in breast cancer MDA-MB-231 cells impaired migration (Xie *et al*. 2013) whereas Smurf 2 knockdown in these cells resulted in enhanced migration *in vitro* and metastasis *in vivo* (Jin *et al*. 2009). The ubiquitin ligase ring finger protein 11(RNF11) interacts with Smurf1 & 2, Smad 4 and other ubiquitin ligases to modulate BMP signalling pathways and both mRNA and protein have been found at high levels in breast tumours (Azmi & Seth 2005).

Further regulation is provided by secreted extracellular BMP antagonists (Walsh *et al*. 2010). BMP antagonists can block the binding of BMPs to their receptors by directly binding to the BMP ligands. These antagonists are often BMP transcription target genes, forming an important regulatory feedback loop for normal tissue development (Alarmo & Kallioniemi 2010). In developing breast epithelium, the interplay between BMPs 2 and 4 and antagonist Noggin is essential for normal ductal elongation and myoepithelial compartmentalisation (Cowin & Wysolmerski 2010, Forsman *et al*. 2013). Noggin, Chordin and Gremlin appear to be upregulated (but not mutated) in breast cancer, but their potential role in breast tumourigenesis has not been well studied and in other cancers they are both pro and anti-tumourigenic (Walsh *et al*. 2010, Owens *et al*. 2015). Finally, Betaglycan (TGF-β receptor III) binds BMPs 2, 4, 7 and GDF5, helping to mediate BMP signalling. Its expression in breast cancer models suppresses BMP-induced invasion and migration through ligand sequestration when in soluble form (Gatza *et al*. 2014).

BMP signalling is heterogeneous and complex, with multiple regulatory influences that are currently implicated in breast cancer, but not yet fully elucidated.

## BMPs in breast tumourigenesis

### Influence of BMPs in cell cycle and proliferation

BMPs are able to regulate the growth of breast cancer cell lines (Table 1). BMP-2, BMP4, BMP-6, BMP-9, BMP-10, BMP-15 and GDF9a impede the proliferation of breast cancer cells (Hanavadi *et al*. 2007, Du *et al*. 2008, Alarmo & Kallioniemi 2010, Ye *et al*. 2010, Ren *et al*. 2014*a*). Inhibition of BMP signalling dramatically downregulates protein levels of mitotic checkpoint components BUB3, Hec1, TTK and MAD2, leading to cell division and tumourigenesis, whereas an upregulation of BMP signalling has the converse effect in breast cancer cells (Yan *et al*. 2012).

**Table 1:** BMP in breast cancer.

	**Expression in breast cancer**		**Function in breast cancer cells**				**Effect** ***in vivo***	
	Primuary tumour	Bone metastases	Proliferation	Apoptosis	Motility	EMT	Primary	Bone
BMP-2	↓/↑		↓	↓/↑	↑	↑	↑	↑
BMP-4	↑		↓/↑		↓/↑	↑	↓/↑	
BMP-5	↑							
BMP-6	↓/↑		↓	↓	↓	↓		
BMP-7	↓/↑	↓/↑	↓/↑		↑	↓	↓	↓
GDF9A	↓		↓		↓			
BMP-9			↓	↑	↑		↓	↓
BMP-10	↓		↓		↓			
BMP-15	↓		↓		↓			
BMPR-IA		↑	↑		↑	↓/↑	↑	↑
BMPR-IB	↓/↑		↓		↑			
BMPR-II	↑		↑		↓			
NOGGIN		↑						↓

Expression of BMPs in breast cancer shown in the table are based on literature: BMP-2 (Soda *et al*. 1998, Clement *et al*. 2000, Ghosh-Choudhury *et al*. 2000*a*,*b*, Pouliot & Labrie 2002, Reinholz *et al*. 2002, Clement *et al*. 2005, Raida *et al*. 2005*a*, Katsuno *et al*. 2008); BMP-4 (Alarmo *et al*. 2007, 2013, Ketolainen *et al*. 2010, Guo *et al*. 2012, Ampuja *et al*. 2013, 2016, Owens *et al*. 2013, Cao *et al*. 2014); BMP-5 (Bobinac *et al*. 2005, Davies *et al*. 2008); BMP-6 (Clement *et al*. 1999, Yang *et al*. 2007, 2009, Du *et al*. 2008, 2009, Lian *et al*. 2013, Hu *et al*. 2016); BMP-7 (Schwalbe *et al*. 2003, Alarmo *et al*. 2006, 2007, 2008, 2009, Buijs *et al*. 2007, Sakai *et al*. 2012); GDF9A/BMP-15 (Hanavadi *et al*. 2007); BMP-9 (Wang *et al*. 2011, Ren *et al*. 2014*a*,*b*); BMP-10 (Ye *et al*. 2010); BMPR-IA (Katsuno *et al*. 2008, Pickup *et al*. 2015*a*); BMPR-IB (Helms *et al*. 2005, Bokobza *et al*. 2009, Allison *et al*. 2016); BMPR-II (Pouliot *et al*. 2003, Owens *et al*. 2012); Noggin (Tarragona *et al*. 2012). Up arrows indicate upregulated (expression) or promote (function and effect), whilst the down arrows indicate downregulated (expression) or inhibit (function and effect).

Studies have focused on the effects of BMP-2, which has a direct anti-proliferative effect on tumour cells at a very high concentration *in vitro* (Soda *et al*. 1998). A kinase inactive type II TGF-β receptor (dnTbetaRII) eliminated the anti-proliferative effect of BMP-2 in breast cancer cells by preventing the phosphorylation of Smad-1 (Dumont & Arteaga 2003). Interestingly, Waite and coworkers demonstrated that BMP-2 increases PTEN expression in MCF-7 cells with resultant decreased proliferation. PTEN is a tumour suppressor affecting proliferation by modulating the PI3K/Akt pathway. Mutations in PTEN are associated with Cowden’s disease, in which there is a markedly increased risk of breast carcinoma. In the presence of BMP-2, association of PTEN with ubiquitin conjugating proteins was reduced, indicating BMP-2 may decrease PTEN degradation, thus increasing the pool of available PTEN and resulting in inhibition of cellular proliferation (Waite & Eng 2003). The presence and composition of BMP receptor complexes can also influence the effect of BMPs on breast tumourigenesis, as although BMPR-IB mediates an inhibition of breast cancer proliferation, BMPR-IA (ALK-3) contributes to progression of breast cancer at primary and secondary sites (Katsuno *et al*. 2008). BMPR-II promotes BMP-induced proliferation in breast cancer cells (Bokobza *et al*. 2009) and over-expression of a dominant negative BMPR-II in T-47D breast cancer cells led to an arrest of cancer cells at the G1 phase of the cell cycle (Pouliot *et al*. 2003).

With regard to cell cycle, it appears that many BMPs have direct and indirect anti-proliferative effect in breast cancer, but this may be subject to aberrations in the balance or function of BMP receptors.

### Influence of BMPs on apoptosis

BMP-2 under routine culture conditions, shows pro-apoptotic effect in MCF-7 breast cancer cells, in which the expression and function of apoptosis related genes, particularly protein kinase R (PKR), and subsequent activation of its substrate eIF2α are regulated by BMP signalling (Steinert *et al*. 2008). In MDA-MB 231 cancer cells, overexpression of Neogenin (a co-receptor for BMPs) significantly increased apoptosis whilst inhibiting BMP-2 induced phosphorylation of Smad1/5/8. This interesting new player appears to modulate BMP/Smad signalling, resulting in the observed effect on apoptosis (Zhang *et al*. 2015). The exact mechanism is not yet known, but Neogenin forms a complex with repulsive guidance molecules (RGM) (Bell *et al*. 2013), such as RGMB, which are also co-receptors for BMP signalling. In MDA-MB-231 cells, knockdown of RGMB promoted survival, reduced Caspase 3 expression and promoted growth and migration via regulation of Smad dependant and independent BMP signalling (Li *et al*. 2012).

However, under different experimental conditions, without supplement of serum, BMP-2 increases the resistance of MCF-7 breast cancer cells to hypoxia-induced apoptosis, via the activation of both the MAPK pathway and ID-1, and suppression of Caspase-3 (Clement *et al*. 2000, Raida *et al*. 2005*a*). The other example is BMP-6, which inhibits proliferation through an upregulation of miRNA-192 and resultant repression of cell cycle progression in MDA-MB-231 cells (Du *et al*. 2008). However, under deprivation of serum, BMP-6 protects MDA-MB-231 cancer cells from stress-induced apoptosis through upregulation of survivin, via the Smad dependent pathway, and activation of p38 via the Smad-independent pathway, with both contributing to the anti-apoptotic effect of BMP-6 (Du *et al*. 2008).

It appears with regard to apoptosis in breast cancer that BMPs may have a dual role dependent on cellular conditions, being pro-apoptotic unless under conditions of cellular stress. However, the evidence from *in vitro* study warrants further exploration for their possible role in therapeutic resistance.

### Expression of BMPs and clinical correlations

In clinical breast cancer samples, decreased mRNA expression of BMP-2, BMP-7, BMP-10 and GDF-9a (an analogue of BMP-15/GDF-9b) were seen and associated with poor clinical outcomes (Reinholz *et al*. 2002, Buijs *et al*. 2007, Hanavadi *et al*. 2007, Davies *et al*. 2008, Ye *et al*. 2010). In contrast, BMP-2, BMP-4, BMP-5 and BMP-7 expression has been reported as elevated in breast tumours and the latter two associated with poor prognosis (Bobinac *et al*. 2005, Raida *et al*. 2005*a*, Alarmo *et al*. 2006, 2007, Davies *et al*. 2008). The key may be that BMPs have bidirectional actions in breast cancer, such as BMP-4, which not only suppresses breast cancer cell growth, but also promotes invasion and migration. Immunohistochemistry studies associated BMP-4 expression with low proliferation tumours, but also increased recurrence (Alarmo *et al*. 2013). Interestingly, this may be supported by the finding of increased transcript levels of BMP-4 and its receptor BMPRII in the peripheral blood of breast cancer patients in advanced disease (Gul *et al*. 2015). Another potentially bidirectional BMP, both increased and decreased BMP-7 expression in primary breast tumours has been correlated with disease-specific bone metastases (Buijs *et al*. 2007, Alarmo *et al*. 2008).

The difference in findings from clinical samples may reflect the heterogeneity of breast cancer and the crosstalk of BMP signalling with a variety of other signalling pathways critical in breast tumourigenesis.

## BMPs and clinical subtypes of breast cancer

It has become clear that breast cancers are heterogeneous, with distinct subtypes based on molecular profile (Cancer Genome Atlas Network 2012), and clinically, treatments are increasingly directed toward molecular markers such as the hormone receptors. The development of hormonal therapies confirmed a distinction in behaviour between oestrogen receptor (ER)-positive and ER-negative breast cancers. Tamoxifen was initially used as a treatment for all breast cancers, but it later became apparent that only those tumours expressing hormone receptors benefit. The introduction of trastuzumab (Herceptin) has also introduced tumour profiling of HER2 expression as a standard in clinical care. More recently, gene expression profiling has made its way into the clinic to predict those with early breast cancer at risk of relapse that would benefit from chemotherapy. Receptor status appears to influence the effect of BMP signalling as seen in clinical studies and several *in vitro* studies, which could make BMP/BMPR status another important profiling marker.

### BMPs and oestrogen receptor signalling

Oestrogen regulates the expression of BMPR-IA, BMPR-IB, ActRIIA and ActRIIB, but has no effect on the expression of ActR1 and BMPR-II (Takahashi *et al*. 2008). Elevated expression of BMPR-IB was associated with high tumour grade, high tumour proliferation, cytogenetic instability and a poor prognosis in ER positive carcinomas (Helms *et al*. 2005). A decreased level of BMPR-IB associated with poor prognosis in a majority of ER-negative tumours (Bokobza *et al*. 2009).

The expression of BMP-7 highly correlates with the expression level of ER, although BMP-7 expression reduces in response to oestrogen (Schwalbe *et al*. 2003, Alarmo & Kallioniemi 2010). BMP-2 expression is significantly higher in the ER-negative tumours (Julien *et al*. 2011). Silencing of ERα results in resistance to effects of oestradiol increased BMP-2 expression, and genetic changes associated with epithelial–mesenchymal transition (EMT) (Al Saleh *et al*. 2011). BMP-6 mRNA has been declared both increased and reduced in comparison with non-tumour margins (Clement *et al*. 1999). Studies show over-expression of BMP-6 particularly in ER positive cell lines and tumour samples (Ong *et al*. 2004, Zhang *et al*. 2007), however, BMP-6 inhibits oestrogen-induced mitosis of ER positive breast cancer cells (Takahashi *et al*. 2008) and targeting BMP-6 in MCF-7 cells using shRNA knockdown promoted cell proliferation (Lian *et al*. 2013).

The mechanism of these interactions is being explored. BMP-6 appears to be activated in a dose- dependant manner by oestrogen through interaction of ER with sites on the BMP-6 promoter region (Zhang *et al*. 2005). In addition, BMP-6 promoter methylation status correlates with ER status in breast cancer. Methylation of the BMP-6 gene promoter has been detected in ER-negative MDA-MB-231 cells; whereas in ER positive MCF-7 and T47D, the BMP-6 gene promoter remains demethylated. In 33 breast tumour specimens, hypermethylation of BMP-6 was observed in all ER-negative cases whereas lower methylation frequency was observed in ER positive cases (Zhang *et al*. 2007).

Oestrogen interferes with the biological function of BMP-2 by inhibiting the activation of Smad, as a result of biochemical interaction between Smad and ERα (Yamamoto *et al*. 2002). Smad-4 can associate with cytoplasmic ERα, preventing the transcriptional regulation mediated by ERα (Wu *et al*. 2003). In MDA-MB-231 cells BMP-2 treatment induced the expression of a splicing variant of ER (ERα-36) in a dose-dependent manner, and growth of MDA-MB-231 cells could be stimulated by oestradiol, even though they were insensitive to it before BMP-2 induction. When the BMP-2 signalling pathway was silenced by si-BMPRIA and si-BMPRIB, the ERα-36 induction was eradicated (Wang *et al*. 2012).

In a bidirectional manner, BMPs as well as being affected by ER signalling can in turn have an effect on ER signalling. BMP-2 inhibits oestradiol-induced proliferation of breast cancer cells, via upregulation of cyclin kinase inhibitor p21, which in turn inhibits the oestradiol-induced cyclin D1-associated kinase activity (Ghosh-Choudhury *et al*. 2000*a*). The Smad dependent signalling is indispensable for BMP-2 induced p21 expression and the consequent inhibitory effect on cell proliferation (Pouliot & Labrie 2002).

It is evident that the ER status has a bearing on the cells’ response to BMPs and vice versa, at both nuclear and cytoplasmic level, involving signalling cross talk and transcriptional regulation. Once again it is likely that the resultant influence on breast tumours and their response to hormonal therapies depend on the balance of these interactions between the signalling pathways.

### BMPs and other signalling crosstalk

Co-regulation of the growth of breast cancer cells can occur between the BMP and other cell signalling pathways. This includes other members of the TGF-β super family (Katsuno *et al*. 2008), epidermal growth factor (EGF) (Schwalbe *et al*. 2003), hepatocyte growth factor (HGF) and HGF receptors (Imai *et al*. 2005, Ye *et al*. 2007*a*, 2008) and Wnt signalling (Guo & Wang 2009).

The upregulation of p21 by BMP-2 prevents EGF-induced proliferation of MDA-MB-231 breast cancer cells (Ghosh-Choudhury *et al*. 2000*b*). This may reflect why MDA-MB-231, an ER-negative tumour cell line, responds to recombinant human BMP-2 with a more significantly reduced proliferation, in comparison with the ER positive MCF-7 cells (Arnold *et al*. 1999).

BMP-4 is considered as an inhibitor of breast cancer cell growth, but can also have a synergistic effect on proliferation of breast cancer cells induced by fibroblast growth factor (FGF), EGF and HGF (Montesano *et al*. 2008). EGF treatment of breast cancer cells *in vitro* upregulated BMP-4 signalling via the Smad pathway, leading to suppression of matrix metalloprotease (MMP) 9. This suppression was attenuated with an addition of BMP-4 antagonist Gremlin or Smad 6 (Laulan & St-Pierre 2015). In addition, BMP-6 in breast cancer cells can be upregulated by EGF and other EGFR ligands such as transforming growth factor-α, amphiregulin and betacellulin (Clement *et al*. 1999). Conversely, EGF, FGF and HGF activated MAPK/ERK results in a phosphorylation of the linking region of Smad1/5/8 leading to a reduced nuclear translocation and a suppression of BMP target genes (Kretzschmar *et al*. 1997, Guo & Wang 2009). BMPs exert reciprocal effects, suppressing EGF-induced gene transcription through MAPK/ERK-1 signalling (Ghosh Choudhury *et al*. 1999). BMP-9 inhibits the proliferation and metastasis of SK-BR-3 breast cancer cells via decreasing HER2 expression and inactivating ERK1/2 and PI3K/AKT signalling pathways (Ren *et al*. 2014*a*).

SOSTDC1, a secreted regulator of both BMP and Wnt signalling pathways, is under expressed in breast cancer and can differentially affect signalling induced by Wnt3a, BMP-2 and BMP-7. In breast cancer cells, SOSTDC1 modestly increases Wnt3a signalling, decreases BMP-7 signalling, whilst eliciting little effect on BMP-2-induced signalling (Clausen *et al*. 2011).

This highlights the important influence of other signalling pathways and the canonical or noncanonical BMP signalling pathways, and may be one of the reasons for the varied and sometimes contradictory study outcomes regarding BMPs in breast cancer.

### BMPs and the androgen receptor

More recently androgen receptor status has become a focus of research, particularly in relation to treatment resistance. Reported as either tumour suppressor or promoter, its expression has been linked to both good and poor prognosis (Feng *et al*. 2017). In ER positive tumours that respond to neoadjuvant endocrine therapy, AR mRNA and protein expression decreases, whereas in tumours those fail to respond, AR mRNA does not decrease. AR over-expression increases tamoxifen resistance in breast cancer models *in vitro* and *in vivo*. In a clinical cohort, a high AR: ER ratio was shown as an independent risk for failure of tamoxifen treatment and poor survival (Cochrane *et al*. 2014).

Upon an ERK-mediated phosphorylation, BMP-activated Smad1 can bind to AR leading to an inhibition of AR-induced transcription and its corresponding effect on cellular functions of prostate cells (Guo & Wang 2009). It is not yet known whether similar interactions between BMP signalling and AR are found in breast cancers, and this would be a novel area of exploration and possible targeted therapy for endocrine treatment resistant breast cancers.

## BMPs and progression of breast cancer

### BMPs in epithelial–mesenchymal transition (EMT)

EMT is an important event during the development and progression of cancer, causing disruption of epithelial homeostasis that may lead to carcinogenesis; it can also transform the indolent tumour cells into a more aggressive colony, leading to metastasis (Larue & Bellacosa 2005, Lamouille *et al*. 2014). The early steps of metastasis, such as invasion and extravasation are facilitated by cells acquiring mesenchymal traits, however, the ability to colonise distant tissues and form macroscopic metastases may be facilitated more by epithelial properties, and thus the breast cancer cell at any one time may be triggered towards either EMT or MET by differential BMP signalling.

EMT regulated by BMPs has been implicated in foetal and postnatal development of different organs and tissues, including mammary gland development, where BMP-2 and 4 have essential roles in both epithelial and mesenchymal differentiation (Nakajima *et al*. 2000, Romano & Runyan 2000, Hens & Wysolmerski 2005).

**Figure 2:**
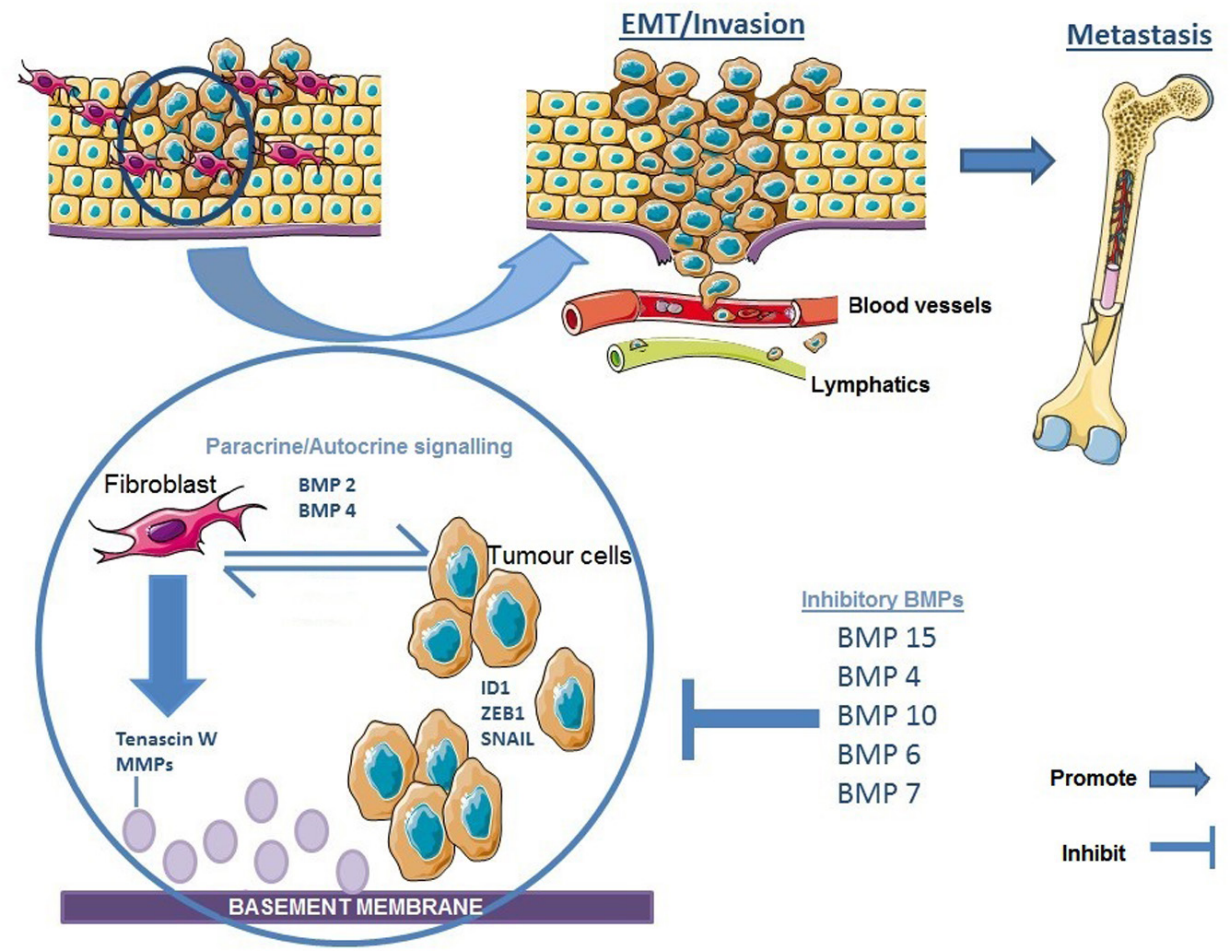
BMPs mediate crosstalk between breast cancer cells and microenvironment during disease progression. BMP signalling by tumour-associated stromal cells (or cancer associated fibroblasts) alter the expression of EMT-related genes in breast cancer cells such as ZEB1, with BMP-2 and 4 in particular, promoting the mesenchymal phenotype. Likewise, breast cancer cells secreting BMP-2 and 4 can stimulate fibroblasts to produce chemokines and enzymes (Tenascin W, MMPs) that promote invasion and motility. Breast cancer cells are then able to disseminate to bone via lymphovascular invasion. However, there are BMPs that reduce invasion (BMP-6, BMP-10, BMP-15) and others who have a dual or bidirectional role dependant on cellular context, such as BMP-4. The figure was created using Servier Medical Art tools http://servier.com. A full colour version of the figure is available at http://dx.doi.org/10.1530/ERC-17-0139

*In vitro,* BMP-4 subverts the ability of mammary epithelial cells to form polarized lumen-containing structures, and also endows them with invasive properties, demonstrating a direct effect promoting a mesenchymal phenotype (Montesano 2007). TGF-β and BMP-2 signalling in murine mammary cancer cell lines results in transcription of genes that suppress the epithelial phenotype. miR-200 counteracts this by targeting the BMP-2 downstream transcription factors responsible for epithelial gene repression, such as Crtap, Fhod1, Smad2, Map3k1, Tob1, Ywhag/14-3-3γ, Ywhab/14-3-3β, Smad5, Zfp36, Xbp1, Mapk12 and Snail (Perdigao-Henriques *et al*. 2016). BMP-2 appears to promote motility and invasiveness of MCF-7 and MDA-MB-231 cells, both *in vitro* and *in vivo* (Clement *et al*. 2005, Katsuno *et al*. 2008). BMP-2 upregulation of target gene ID-1 (which activates pathways involved in tumour progression) may contribute to this effect (Gautschi *et al*. 2008) (Fig. 2).

BMP receptors are also important in EMT, with application of a type I BMPR inhibitor to mice reducing key EMT-related genes such as Snail, Twist, Zeb1 and Zeb2 (Balboni *et al*. 2013, Owens *et al*. 2015). In humans, high BMPRIA expression correlates with poor survival (Pickup *et al*. 2015*a*). Knockdown of BMPRIA *in vivo* delayed tumour onset, and also subsequent growth of tumours and improved survival, despite conversely seeming to induce EMT-like tumour transitions, such as increased Vimentin (Pickup *et al*. 2015*a*).

Not all BMPs induce EMT, and some appear to promote MET, reducing the aggressive properties of tumour cells. In murine mammary epithelial cells (NMuMG), BMP-7 was not able to induce EMT whereas TGF-β1 could (Piek *et al*. 1999). BMP-7 is able to increase cytokeratin expression, and decrease vimentin in breast cancer cells *in vitro* and *in vivo*, leading to an epithelial-like phenotype (Buijs *et al*. 2007). This effect is also seen with BMP 6, which restores E-cadherin-mediated cell-to-cell adhesion and prevents breast cancer metastasis through the downregulation of miR-21 and δEF1 (ZEB1, whose expression associates with invasive breast cancer phenotype) (Yang *et al*. 2007, Du *et al*. 2009, de Boeck *et al*. 2016).

### BMPs effect on tumour microenvironment, migration and invasion

Tumour microenvironment and the interaction between tumour cells and surrounding support cells are important for the progression and invasion of tumours (Fig. 2). Stimulation of fibroblasts by BMP signalling can promote breast tumour cell invasion and increased inflammatory cytokine production (Owens *et al*. 2012). Loss of BMPRII in murine fibroblasts promoted tumour metastasis and sustained inflammatory cell infiltration (Pickup *et al*. 2015*b*). This suggests BMPRII can have both direct suppressive effects on tumour cells, but also indirectly via regulation of inflammation in the tumour-associated stroma (Owens *et al*. 2012, Pickup *et al*. 2015*b*).

In triple negative MDA-MB-468 cells, upregulated BMPRIB showed increased migratory capacity in response to BMP-2, which was abrogated by the BMPR antagonist dorsomorphin (Allison *et al*. 2016). An analogue of dorsomorphin (DMH1), much more highly selective for type 1 BMPR, can attenuate the pro-tumour microenvironment by altering the expression of certain genes (such as ID-1 and matrix metalloproteases-MMPs) in fibroblasts, lymphatic vessels and macrophages in a mouse model (Owens *et al*. 2015).

BMP-2 may contribute to the invasiveness of tumour cells via induction of the extracellular matrix glycoprotein Tenascin-W in the tumour-surrounding stroma. Smad-independent signalling through p38 and JNK pathways is involved in BMP-2 induction of Tenascin-W and overexpression of Tenascin-W in the stroma of breast cancer promotes invasion and migration of cancer cells through an interaction with α8 integrin (Scherberich *et al*. 2005).

Treatment with BMP-4 increased invasion and migration in both breast cancer cell lines and a mouse model (Ketolainen *et al*. 2010, Guo *et al*. 2012, Ampuja *et al*. 2013, 2016). CCN6 is an extracellular matrix associated protein that has been shown *in vitro* and *in vivo* to directly antagonise this BMP-4 mediated invasiveness and metastases (Pal *et al*. 2012). A similar effect to BMP-2 on stromal cells appears to be true with BMP-4 treatment in mammary stromal fibroblasts. Fibroblasts stimulated with BMP-4 enhanced MCF-7 cell invasion, and these effects were inhibited by DMH1. BMP-4 increased MMP-3 and IL-6 in conditioned medium from treated mammary fibroblasts, suggesting BMP-4 can influence the tumour microenvironment to promote breast cancer invasion (Owens *et al*. 2013).

Interestingly, BMP-4 inhibits aggressiveness in different breast cancer cell lines under different experimental conditions. Overexpression of N-myc downstream-regulated gene 2 (NDRG2) in MDA-MB-231 cells induced BMP-4 and inhibited expression of MMP-1, -3 and -9 compared to control. When BMP-4 was neutralised with anti-BMP-4 antibody, MMP-9 expression recovered and migratory capacity of the cells increased. Application of rhBMP-4 to wild type MDA-MB-231 cells suppressed MMP-9 expression and activity, reducing migration and invasion (Shon *et al*. 2009). Additionally, in a mouse model, BMP-4 suppressed metastasis, seemingly by regulating anti-tumor immune responses (Cao *et al*. 2014).

Forced expression of GDF-9a/BMP-15 in breast cancer cells also reduced invasiveness *in vitro*, as does BMP-10 (Hanavadi *et al*. 2007, Ye *et al*. 2010). Hu and coworkers showed that BMP-6 markedly downregulated matrix metalloproteinase-1 (MMP-1) expression at both the mRNA and protein levels in MDA-MB231 cells, inhibiting invasion, and this effect was significantly attenuated by overexpression of MMP-1. BMP-6 also increased adhesion and cell-cell contacts in these cells (de Boeck *et al*. 2016, Hu *et al*. 2016).

The above experimental evidence suggests that BMPs can differentially influence tumour invasion by regulating the balance of MMPs, extracellular matrix components, cytokines and immune or inflammatory cells in the tumour microenvironment.

### BMPs and angiogenesis in breast cancer

**Figure 3:**
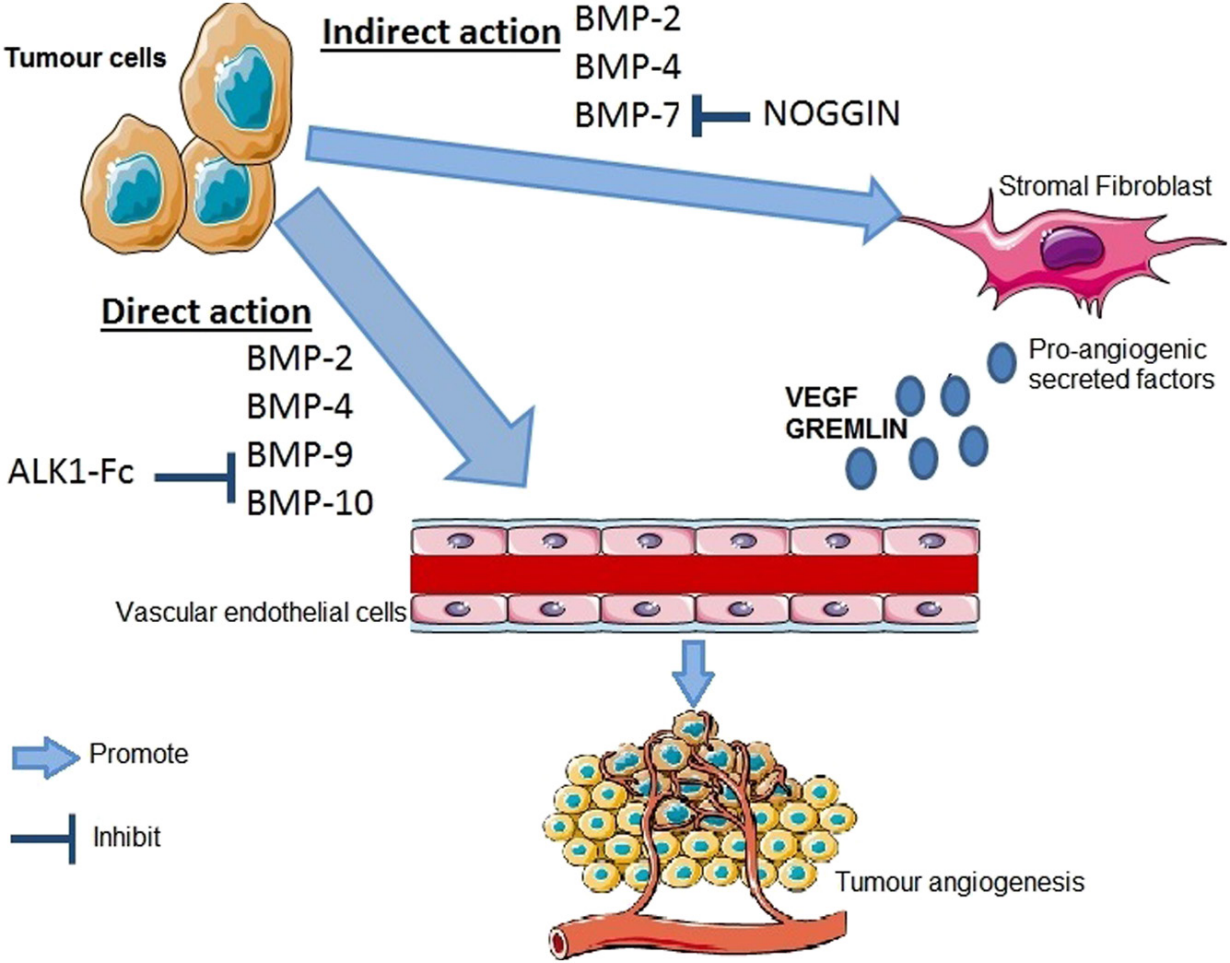
It has been demonstrated that BMP-2, -4, -6, -7, and GDF5, are capable of directly inducing angiogenesis in vascular endothelial cells, whilst effect of BMP 9/10 seems more context dependent. BMP-9 has biphasic effects on endothelial cells. High doses can inhibit endothelial proliferation and migration, whereas low doses can have stimulatory effects. Indirectly, BMPs can act on stromal cells to promote the secretion of pro- angiogenic factors such as VEGF. This can be attenuated by BMP antagonism such as Noggin attenuating the pro-angiogenic effect of BMP 7. Another BMP antagonist, gremlin has a BMP independent function promoting angiogenesis by interaction with VEGF receptors. The figure was created using Servier Medical Art tools http://servier.com. A full colour version of the figure is available at http://dx.doi.org/10.1530/ERC-17-0139

Tumour angiogenesis has been shown to be important in breast cancer progression and metastasis (Ribatti *et al*. 2016) and current knowledge regarding the role of BMPs and angiogenesis has been well reviewed here (Ye & Jiang 2016) (Fig. 3). In general, experimental evidence suggests that BMPs promote angiogenesis indirectly through upregulation of the expression of vascular endothelial growth factor (VEGF) (Yeh & Lee 1999, Deckers *et al*. 2002, Dai *et al*. 2004).

Regarding breast cancer specifically, one study has reported that BMP-2 promotes breast tumour related angiogenesis through stimulating p38 MAPK pathway and ID-1 expression (Raida *et al*. 2005*b*). Conversely, Chi and coworkers demonstrated overexpression of BMP antagonist Coco (DAND5) in breast cancer cells promoted micro-vascular formation *in vitro* and in mouse xenograft tumours, although the mechanism has not been clarified. They also found Coco positivity in breast cancer patient serum correlated with relapse and poor survival, although this could be due to its influence on other aspects of tumour progression (Chi *et al*. 2016). Current ongoing clinical trials are examining the effect of BMP-9 and 10 blocking agents as anti-angiogenic treatments for solid tumours, which is further addressed below. The limited literature regarding BMPs in breast tumour angiogenesis makes this a rich area for study, particularly considering the potential therapeutic applications.

## BMPs and dissemination of breast cancer to bone

### BMPs and the bone environment

A now traditional view of cancer metastasis is Paget's ‘seed and soil’ hypothesis, namely that the cancer cell will deposit and grow only if the environment is favourable towards that cancer cell, i.e., both the seed and the soil have to be mutually compatible. Bone metastasis involves cancer cell dissemination from the primary tumour, extravasation into the blood stream and occupation of the bone marrow space. As vital regulators of bone formation, BMPs have been of great interest in this field with several studies examining their role in bone metastases. In normal bone physiology and turnover, BMP signalling is essential for differentiation of mesenchymal stem cells (MSCs) and maturation into chondroblasts and osteoblasts, resulting in bone formation. BMP-2, BMP-4, BMP-6, BMP-7, BMP-9, BMP-12 and BMP-13 induce MSCs differentiation, but not all are osteoinductive in nature (Alarmo & Kallioniemi 2010, Carreira *et al*. 2014). BMPs also induce osteoblasts to produce certain factors influential to osteoclast maturation and function. Thus, BMPs are an integral part of the bone environment (Alarmo & Kallioniemi 2010, Rahman *et al*. 2015).

### Aberration in BMPs and bone metastasis

In breast cancer, BMP-induced transcriptional pathways are active in bone metastatic lesions *in vivo* and dominant negative BMP receptors reduced bone metastases *in vivo* (Katsuno *et al*. 2008). Decreased expression of BMP-7 in primary tumours correlates with bone metastases and BMP-7 is able to inhibit the growth of breast cancer tumours in bone *in vivo* (Buijs *et al*. 2007). Conversely, other studies have shown BMP-7 overexpression in primary tumours associated with bone metastases (Alarmo & Kallioniemi 2010). In murine 4T1E/M3 mammary cells, which are highly metastatic to bone, expression of BMP-7, BMPR and phosphorylated Smad1/5/8 is upregulated. These highly invasive features are attenuated when BMP-7 is inhibited (Sakai *et al*. 2012). BMP-9 inhibits the growth of breast cancer cells *in vitro* and *in vivo*, and also suppresses the growth of tumour cells in bone (Wang *et al*. 2011, Ren *et al*. 2014*b*). Downregulation of connective tissue growth factor (CTGF) by BMP-9 is involved in the inhibition of tumour growth in bone (Ren *et al*. 2014*b*).

Breast cancer cells themselves can acquire an osteoblast-like phenotype, by ectopically expressing bone matrix proteins such as bone sialoprotein (BSP), osteopontin (OPN), osteoprotegerin (OPG) and osteoblast-specific cadherins (Ibrahim *et al*. 2000, Kapoor *et al*. 2008, Tan *et al*. 2016). Tan and coworkers (Tan *et al*. 2016) showed that breast cancer cells with induced EMT exhibited an elevated level of bone-related genes (BRGs) and osteoblast-like features in an exposure to BMP-2. Breast cancer cells expressing these BRGs preferentially metastasise and survive in bone. It also interestingly made cells more resistant to chemotherapy. These effects were reversed with Noggin application, or knockdown of runt-related transcription factor 2 (RUNX2), which regulates bone remodelling, and osteogenic differentiation (Alarmo & Kallioniemi 2010, Carreira *et al*. 2014, Tan *et al*. 2016). This osteomimicry induced by BMPs may be one of the reasons breast cancer cells home to bone tissue and survive in the bone microenvironment (Rucci & Teti 2010). In other words, BMP signalling in breast tumours could create a subset of bone-specific metastatic cells: the right kind of ‘seed’ for a specific soil.

### Regulators of BMP signalling in bone metastases

BMPs and their antagonists can also influence the bone microenvironment, for example, orthotropic implant of silk scaffolds carrying BMP-2 showed increased metastatic spread of breast cancer cells to bone *in vivo* (Moreau *et al*. 2007). Conditioned medium (CM) from HT-39 breast cancer cells promoted osteoblastic behaviour in osteoprogenitor cells. This effect was blocked by addition of Noggin (Bunyaratavej *et al*. 2000). High expression levels of Noggin are associated with bone metastases in both cell line/murine models and clinical samples of breast cancer bone metastases (Tarragona *et al*. 2012). Upregulation of Noggin and Follistatin by ZEB1 in breast cancer cells induced differentiation of osteoclasts *in vitro*, suggesting an osteolytic influence in the bone microenvironment (Mock *et al*. 2015), however the role BMP antagonists play in coordinating the osteoblastic and osteolytic activities in bone metastatic lesions are far from being clear.

Regulation of BMP signalling by oestrogen and ER may also contribute to osteoblast differentiation and thus may influence the bony metastatic niche. The selective estrogen receptor modulator raloxifene increased the activity of the BMP-4 promoter in U-2 OS osteoblast-like cells. ER-α is thought to be indispensable for this effect on the BMP-4 promoter and may be part of the mechanism of this agents in reducing both osteoporosis and breast cancer risk (van den Wijngaard *et al*. 2000).

Oestradiol enhances BMP-4-induced expression of osteoblastic markers (Runx2, osterix, osteocalcin) in osteoprogenitor cells. In contrast, the expression of ER-α and endogenous BMP-4 was suppressed by BMP-4 treatment regardless of the presence of oestrogen, implying the presence of a negative feedback loop for osteoblast differentiation (Matsumoto *et al*. 2013).

In osteoblasts, BMP- 6 reporter activity increased with anti-oestrogen treatment, and decreased with oestradiol treatment, providing evidence that ER regulates BMP-6 differentially in breast and bone, and ERα-dependent pathways (such as BMPs) may influence skeletal secondary formation in breast cancer, which is consistent with the previous observation that patients with ER positive breast tumours are more likely to develop skeletal metastases (Ong *et al*. 2004).

In summary, BMPs and their regulators, such as BMP antagonists or ER signalling, can also result in a bone environment receptive and supportive of metastases, and influential in the balance between osteogenesis and osteolysis, i.e., they affect the bone ‘soil’ conditions.

## BMPs and breast cancer relapse

As a consequence of improvements in breast cancer treatments, nearly 80% of women survive at least 10 years after their diagnosis (Cancer Research UK 2017). For women with triple negative disease there is high risk of early recurrence, reflecting the aggressive nature of this subtype, and the lack of targeted treatments. For receptor-positive tumours, the risk of recurrence is lower, but continues potentially for decades after diagnosis (Yamashita *et al*. 2016).

Relapse of disease is often attributed to cancer stem cells, cells with tumour initiating capacity and the ability to evade the effects of chemotherapy by remaining in an alive but quiescent or dormant state, only to clinically manifest at a later point, causing symptoms and death (Oskarsson *et al*. 2014).

### BMPs and breast cancer stem cells

Mammary tissue inevitably contains stem and progenitor cells, undergoing cycles of quiescence and proliferation throughout mammary development, maturation and involution (Woodward *et al*. 2005). Stem cells share many of the characteristics of cancer cells, including the ability to proliferate through a process of self-renewal and a loss of contact inhibition and BMPs seem to play a role in stem cell and progenitor determination.

BMP-2 enhanced production of luminal progenitors in MCF10A mammary cells, whereas BMP-4 prevented differentiation. BMP-4 redirected these cells towards an immature progenitor phenotype, suggesting a balance between BMP-2 and BMP-4 defines mammary cell fate (Clement *et al*. 2017).

In studies with human embryonic stem (hES) cells, BMPs promote differentiation, dependent on the feeder cells on which the hES are grown and in the context of other signalling pathways. For example, in the presence of FGF signalling BMP induces hES cells to differentiate into the trophoblast lineage. In the presence of FGF and BMP antagonist Noggin, hES cells can be maintained in the pluripotent state. This implicates the balance of BMP and antagonists in the switch between states of self-renewal and differentiation (Varga & Wrana 2005).

In breast cancers, the influence of BMP signalling on stem cell populations is not yet clear, and varies dependant on experimental conditions. A BMP2/7 heterodimer strongly reduced the size of a breast cancer stem cell population *in vitro,* and *in vivo* was able to inhibit formation of bone metastases (Buijs *et al*. 2012). Conversely, in separate studies, a BMPR inhibitor reduced stem cell populations and clonogenic capacity in established mammary epithelial cell lines and primary murine tumor cells (Balboni *et al*. 2013).

Autocrine BMP-4 signalling maintained the stem cell phenotype of an A17 invasive mesenchymal cell line, whereas BMP-4 inhibition by dorsomorphin resulted in epithelial-like traits, by downregulating Snail and Slug transcription factors, resulting in loss of stem-features and self-renewal ability (Garulli *et al*. 2014). It may be that differential BMPs and receptor profiles in autocrine and paracrine signalling result in the variety of effect on breast stem cell populations.

### BMPs and quiescence

As well as influencing stem cells, some studies suggest BMPs could induce stem cell quiescence, which would have important implications for disease relapse. When expression of tumour suppressor ΔNp63α was induced in MCF-7 cells, the BMP target gene ID-1 was upregulated and proliferation significantly reduced. There was an increase in proportion of progenitor like cells, and cells in reversible G0 cell phase. The authors suggest BMP signalling induced quiescence in MCF7 cells, mediated by ΔNp63α (Amin *et al*. 2016).

Gao and coworkers demonstrated that paracrine BMP signalling suppresses cancer stem cell traits, and that BMP antagonist Coco reactivates dormant metastatic breast cancer cells in the lungs. Coco induced a self-renewing stem cell-like phenotype in the metastatic cells by blocking the BMP-induced repression of core stem cell transcription factors (Gao *et al*. 2012).

Therapies usually target proliferating cells, thus quiescence in disseminated breast cancer cells can result in evasion of treatment and disease relapse, potentially many years later (Zhang *et al*. 2013). BMP signalling influences both self-renewal of cells and the switch between active proliferation and quiescence. This is a key area for further development in treating, predicting and preventing relapse, which remains a significant clinical challenge in breast cancer.

## Implications in breast cancer therapeutics

BMPs are instrumental in the differentiation of bone marrow mesenchymal stem cells into bone producing osteoblasts in normal bone turnover. BMPs may swing the balance between osteolysis and osteogenesis, as they promote osteoblast differentiation, which directly promotes osteogenesis and is thus the reason recombinant BMP-2 is utilised in orthopaedic surgery. But as BMPs promote the production of receptor activator of nuclear factor kappa-B ligand (RANKL) by osteoblast precursors, this indirectly promotes osteoclastogenesis and bone resorption. In addition, BMP and Wnt pathways are major regulators of normal osteogenesis.

### Targeting Wnt/BMP signalling

**Figure 4:**
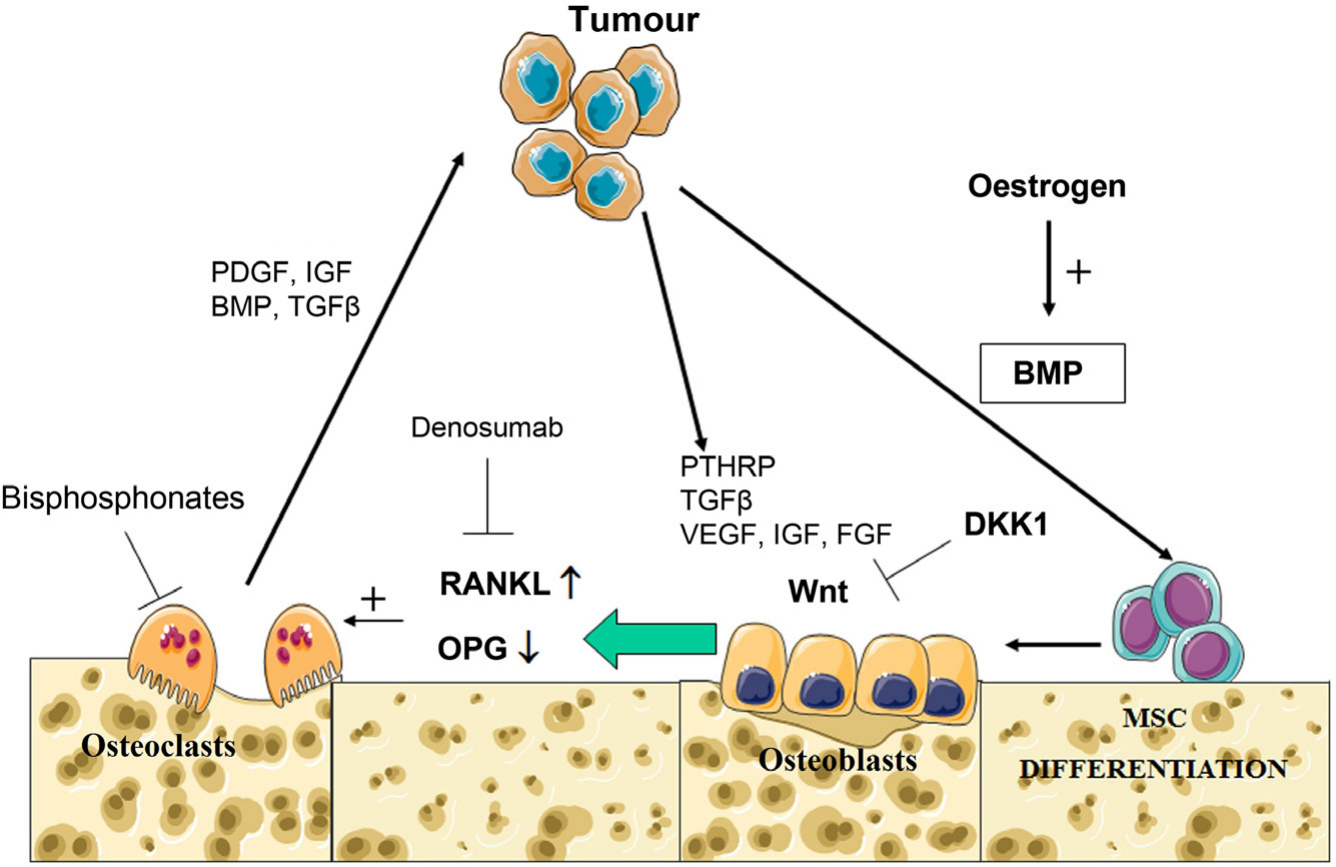
BMPs are a vital component of normal bone turnover, stimulating the differentiation of mesenchymal stem cells (MSC) into osteoblasts, which is promoted by oestrogen. Osteoblasts secrete RANKL and OPG. If the balance of RANKL is higher than its inhibitor OPG, RANKL binds to osteoclast precursors and results in their maturation and resultant osteolysis. In the vicious cycle of bone metastases, tumour cells secrete many factors which encourage osteoblast production of RANKL and downregulation of OPG, resulting in net osteolysis, which further releases factors that support the survival and proliferation of the tumour cells. Wnt signalling encourages osteoblast maturation and OPG secretion. DKK1, downstream of BMP signalling, inhibits Wnt signals, and thus tips the balance towards osteolysis and reduced bone mass, making DKK1 a therapeutic target in osteoporosis and osteolytic bone metastases. In the current management of osteolytic bone lesions, denosumab is a RANKL inhibitor, reducing osteoclast maturation and function, whereas bisphosphonates penetrate the bone environment, binding to calcium and are then taken up by osteoclasts resulting in osteoclast apoptosis. The figure was created using Servier Medical Art tools http://servier.com. A full colour version of the figure is available at http://dx.doi.org/10.1530/ERC-17-0139

The Wnt inhibitors sclerostin and dickkopf 1 (DKK1) act physiologically as downstream molecules of BMP signalling to inhibit canonical Wnt signalling and therefore negatively regulate bone mass. Tumour production of DKK1 and sclerostin is thought to contribute to osteolytic bone lesions (Lipton *et al*. 2009, Chen *et al*. 2012). A DKK1-neutralizing antibody is in clinical trials for multiple myeloma, and sclerostin-neutralizing antibodies have been developed for osteoporosis. Bortezomib is a proteasome inhibitor, which inhibits osteoclast formation and bone resorption while enhancing osteoblastic differentiation and mineralisation *in vitro*. The detailed mechanism is unclear but may result from decreased DKK1. The fact that BMP signalling acts upstream makes BMP antagonism and interaction with Wnt signalling a future area of exploration for bone metastases therapeutics (Lipton *et al*. 2009, Suvannasankha & Chirgwin 2014) (Fig. 4).

### Targeting osteoclast activity

In breast cancer bone metastasis, parathyroid hormone-related peptide (PTHRP) released from tumour cells up-regulates the expression of RANK-L in preosteoblasts, whilst repressing the expression of osteoprotegerin (OPG, which normally acts to inhibit RANK-L function), leading to a stimulation of osteoclasts and consequent bone resorption. Osteoclastic activity in turn increases the production of factors that increase PTHRP production, including TGFβ, insulin-like growth factor (IGF), platelet-derived growth factor (PDGF), and BMPs, and this supports the survival of the tumour (Suvannasankha & Chirgwin 2014, Yardley 2016). The only current treatments for skeletal events in breast cancer are focused on inhibiting osteoclast function, thus reducing bone resorption. Bisphosphonates bind to bone mineral and are then taken up by osteoclasts, resulting in apoptosis. Denosumab is a monoclonal antibody against RANKL, reducing osteoclast differentiation (Steger & Bartsch 2011) (Fig. 4). The use of bisphosphonates and denosumab is currently a palliative measure, because they have not been consistently shown to improve survival or prevent bone metastasis. Moreover, breast cancer patients who develop pathological fracture have a significant 32% increased risk of death relative to patients without a fracture (Lipton *et al*. 2009). Thus, there is a need for developing agents that act to prevent bone metastasis, and BMPs are under explored in this capacity.

### Targeting phosphoinositide-3-kinase-Akt-mTOR pathway

Another area of therapeutic interest more recently is the phosphoinositide-3-kinase (PI3K)-Akt-mTOR pathway: a key mediator of cellular proliferation, apoptosis, migration and angiogenesis, which is commonly activated in breast cancer, conferring resistance to hormonal therapy and trastuzumab. In lung cancer cells, BMP-2 regulates cellular transformation by activating the PI3K/mTOR pathway, which was completely inhibited by the mTOR inhibitor rapamycin. In breast cancer models, BMP-2 has also been shown to induce the proto-oncogene PI3K in osteoblasts to regulate differentiation. mTOR blockade suppresses RANKL and increases OPG secretion by the bone marrow stroma. mTOR inhibitors are part of ongoing trials regarding hormone receptor-positive treatment resistant tumours, although the apparent involvement of PI3K/mTOR in bone makes it of interest for bone metastasis (Royce & Osman 2015, Zhang *et al*. 2016).

### BMP specific inhibitors

The BMP small molecule inhibitors dorsomorphin and LDN 193189 reverse stem-like features in breast cancer cells and reduce invasiveness, and have been used in several breast cancer studies to abrogate BMP signalling. However, as yet, have not been advanced towards further development for clinical testing in malignancy with propensity to metastasise to bone. One agent that directly affects BMP signalling and is in clinical trials for solid tumours is dalantercept. A soluble chimeric ALK 1 receptor-like protein (ALK1-Fc), which displays high affinity binding with BMP-9 and BMP-10, preventing their interaction with the type 1 receptor ALK1. This results in inhibition of angiogenesis and suppresses tumour growth (Hawinkels *et al*. 2016) (Fig. 3). Initial studies show ALK1-Fc decreased metastasis formation in a breast cancer model (Cunha & Pietras 2011). In mice, treatment with ALK1-Fc did not result in decreased tumour size, but seemed to remodel tumour vasculature, with increased perfusion and reduced hypoxia. A temporary improvement of tumour perfusion could result in a better delivery and efficacy of chemotherapy, and indeed, pretreatment with ALK1-Fc made tumours more sensitive to cisplatin, repressing disease progression (Hawinkels *et al*. 2016). BMPs (particularly 9 and 10) may thus have an important role in primary tumour and bone metastases vascular remodelling, and not only angiogenesis itself. Targeting ALK1 and its ligands are the focus of ongoing clinical trials for anti-angiogenic therapies in breast cancer and other solid tumours, the results of which are awaited with interest.

## Conclusions

Considering the prospect of relapsed disease and treatment resistance in breast cancer patients, and the significant burden of skeletal metastases in particular, we currently have only palliative measures for skeletal related events, and no bone-specific predictors, biomarkers or preventative therapies for bone metastases.

Aberrant expression of BMPs and BMP signalling has been implicated in breast cancer and disease-specific bone metastasis. BMPs are, as the evidence suggests, part of a highly complex, contextual and contrary signalling pathway, where balance is key to effect. The more recent studies have demonstrated BMP signalling activity in both breast primary tumours and bone metastases, contributing to EMT, angiogenesis, invasion, stemness and quiescence, bone-related phenotypes, osteogenesis and osteolysis.

These findings collectively indicate a promising therapeutic value for BMPs and their antagonists in the management of bone metastases by influencing the propensity to disseminate to and survive in the bone microenvironment. In altering the balance of bone turnover to reduce osteolysis and the morbidity associated with it, they may also be a useful adjunct to the RANKL inhibitors currently used to palliate osteolytic lesions. The current clinical trials targeting ALK-1 BMP receptors to influence tumour angiogenesis and effectiveness of chemotherapies clearly show that the BMP pathway contains a wealth of potential.

## Declaration of interest

The authors declare that there is no conflict of interest that could be perceived as prejudicing the impartiality of this review.

## Funding

This work did not receive any specific grant from any funding agency in the public, commercial, or not-for-profit sector.

## References

[bib1] Al Saleh S Al Mulla F Luqmani YA 2011 Estrogen receptor silencing induces epithelial to mesenchymal transition in human breast cancer cells. PLoS ONE 6 e20610 (10.1371/journal.pone.0020610) 21713035 PMC3119661

[bib3] Alarmo EL Kallioniemi A 2010 Bone morphogenetic proteins in breast cancer: dual role in tumourigenesis? Endocrine-Related Cancer 17 R123–R139. (10.1677/ERC-09-0273) 20335308

[bib7] Alarmo EL Rauta J Kauraniemi P Karhu R Kuukasjarvi T Kallioniemi A 2006 Bone morphogenetic protein 7 is widely overexpressed in primary breast cancer. Genes, Chromosomes and Cancer 45 411–419. (10.1002/gcc.20307) 16419056

[bib5] Alarmo EL Kuukasjarvi T Karhu R Kallioniemi A 2007 A comprehensive expression survey of bone morphogenetic proteins in breast cancer highlights the importance of BMP4 and BMP7. Breast Cancer Research and Treatment 103 239–246. (10.1007/s10549-006-9362-1) 17004110

[bib4] Alarmo EL Korhonen T Kuukasjarvi T Huhtala H Holli K Kallioniemi A 2008 Bone morphogenetic protein 7 expression associates with bone metastasis in breast carcinomas. Annals of Oncology 19 308–314. (10.1093/annonc/mdm453) 17895257

[bib6] Alarmo EL Parssinen J Ketolainen JM Savinainen K Karhu R Kallioniemi A 2009 BMP7 influences proliferation, migration, and invasion of breast cancer cells. Cancer Letters 275 35–43. (10.1016/j.canlet.2008.09.028) 18980801

[bib2] Alarmo EL Huhtala H Korhonen T Pylkkanen L Holli K Kuukasjarvi T Parkkila S Kallioniemi A 2013 Bone morphogenetic protein 4 expression in multiple normal and tumor tissues reveals its importance beyond development. Modern Pathology 26 10–21. (10.1038/modpathol.2012.128) 22899288

[bib8] Allison SE Chen Y Petrovic N Zimmermann S Moosmann B Jansch M Cui PH Dunstan CR Mackenzie PI Murray M 2016 Activation of the pro-migratory bone morphogenetic protein receptor 1B gene in human MDA-MB-468 triple-negative breast cancer cells that over-express CYP2J2. International Journal of Biochemistry and Cell Biology 80 173–178. (10.1016/j.biocel.2016.10.004) 27720933

[bib113] American Cancer Society 2016 Current grants by cancer type. Atlanta, GA, USA: American Cancer Society (available at: https://www.cancer.org/research/currently-funded-cancer-research/grants-by-cancer-type.html)

[bib9] Amin R Morita-Fujimura Y Tawarayama H Semba K Chiba N Fukumoto M Ikawa S 2016 DeltaNp63alpha induces quiescence and downregulates the BRCA1 pathway in estrogen receptor-positive luminal breast cancer cell line MCF7 but not in other breast cancer cell lines. Molecular Oncology 10 575–593. (10.1016/j.molonc.2015.11.009) 26704768 PMC5423149

[bib11] Ampuja M Jokimaki R Juuti-Uusitalo K Rodriguez-Martinez A Alarmo EL Kallioniemi A 2013 BMP4 inhibits the proliferation of breast cancer cells and induces an MMP-dependent migratory phenotype in MDA-MB-231 cells in 3D environment. BMC Cancer 13 429 (10.1186/1471-2407-13-429) 24053318 PMC3848934

[bib10] Ampuja M Alarmo EL Owens P Havunen R Gorska AE Moses HL Kallioniemi A 2016 The impact of bone morphogenetic protein 4 (BMP4) on breast cancer metastasis in a mouse xenograft model. Cancer Letters 375 238–244. (10.1016/j.canlet.2016.03.008) 26970275

[bib12] Arnold SF Tims E McGrath BE 1999 Identification of bone morphogenetic proteins and their receptors in human breast cancer cell lines: importance of BMP2. Cytokine 11 1031–1037. (10.1006/cyto.1999.0508) 10623428

[bib13] Awolaran O Brooks SA Lavender V 2016 Breast cancer osteomimicry and its role in bone specific metastasis; an integrative, systematic review of preclinical evidence. Breast 30 156–171. (10.1016/j.breast.2016.09.017) 27750106

[bib14] Azmi P Seth A 2005 RNF11 is a multifunctional modulator of growth factor receptor signalling and transcriptional regulation. European Journal of Cancer 41 2549–2560. (10.1016/j.ejca.2005.08.020) 16226459

[bib15] Balboni AL Hutchinson JA DeCastro AJ Cherukuri P Liby K Sporn MB Schwartz GN Wells WA Sempere LF Yu PB 2013 DeltaNp63alpha-mediated activation of bone morphogenetic protein signaling governs stem cell activity and plasticity in normal and malignant mammary epithelial cells. Cancer Research 73 1020–1030. (10.1158/0008-5472.CAN-12-2862) 23243027 PMC3739305

[bib16] Bell CH Healey E van Erp S Bishop B Tang C Gilbert RJ Aricescu AR Pasterkamp RJ Siebold C 2013 Structure of the repulsive guidance molecule (RGM)-neogenin signaling hub. Science 341 77–80. (10.1126/science.1232322) 23744777 PMC4730555

[bib17] Bobinac D Maric I Zoricic S Spanjol J Dordevic G Mustac E Fuckar Z 2005 Expression of bone morphogenetic proteins in human metastatic prostate and breast cancer. Croatian Medical Journal 46 389–396. 15861517

[bib18] Bokobza SM Ye L Kynaston HE Mansel RE Jiang WG 2009 Reduced expression of BMPR-IB correlates with poor prognosis and increased proliferation of breast cancer cells. Cancer Genomics and Proteomics 6 101–108. 19451094

[bib19] Bragdon B Moseychuk O Saldanha S King D Julian J Nohe A 2011 Bone morphogenetic proteins: a critical review. Cellular Signalling 23 609–620. (10.1016/j.cellsig.2010.10.003) 20959140

[bib20] Buijs JT Henriquez NV van Overveld PG van der Horst G Que I Schwaninger R Rentsch C Ten Dijke P Cleton-Jansen AM Driouch K 2007 Bone morphogenetic protein 7 in the development and treatment of bone metastases from breast cancer. Cancer Research 67 8742–8751. (10.1158/0008-5472.CAN-06-2490) 17875715

[bib21] Buijs JT van der Horst G van den Hoogen C Cheung H de Rooij B Kroon J Petersen M van Overveld PG Pelger RC van der Pluijm G 2012 The BMP2/7 heterodimer inhibits the human breast cancer stem cell subpopulation and bone metastases formation. Oncogene 31 2164–2174. (10.1038/onc.2011.400) 21996751

[bib22] Bunyaratavej P Hullinger TG Somerman MJ 2000 Bone morphogenetic proteins secreted by breast cancer cells upregulate bone sialoprotein expression in preosteoblast cells. Experimental Cell Research 260 324–333. (10.1006/excr.2000.5019) 11035927

[bib23] Cancer Genome Atlas Network 2012 Comprehensive molecular portraits of human breast tumours. Nature 490 61–70. (10.1038/nature11412) 23000897 PMC3465532

[bib24] Cancer Research UK 2017 Cancer Statistics for the UK. London, UK: Cancer Research UK (available at: http://www.cancerresearchuk.org/health-professional/cancer-statistics)

[bib25] Cao Y Slaney CY Bidwell BN Parker BS Johnstone CN Rautela J Eckhardt BL Anderson RL 2014 BMP4 inhibits breast cancer metastasis by blocking myeloid-derived suppressor cell activity. Cancer Research 74 5091–5102. (10.1158/0008-5472.CAN-13-3171) 25224959

[bib26] Carreira AC Alves GG Zambuzzi WF Sogayar MC Granjeiro JM 2014 Bone morphogenetic proteins: structure, biological function and therapeutic applications. Archives of Biochemistry and Biophysics 561 64–73. (10.1016/j.abb.2014.07.011) 25043976

[bib27] Chen G Deng C Li YP 2012 TGF-beta and BMP signaling in osteoblast differentiation and bone formation. International Journal of Biological Sciences 8 272–288. (10.7150/ijbs.2929) 22298955 PMC3269610

[bib28] Chi Y Yao L Hu X Huang S Huang N Li S Shao Z Wu J 2016 The BMP inhibitor DAND5 in serum predicts poor survival in breast cancer. Oncotarget 7 14951–14962. (10.18632/oncotarget.7498) 26908452 PMC4924764

[bib29] Clausen KA Blish KR Birse CE Triplette MA Kute TE Russell GB D’Agostino RB Jr Miller LD Torti FM Torti SV 2011 SOSTDC1 differentially modulates Smad and beta-catenin activation and is down-regulated in breast cancer. Breast Cancer Research and Treatment 129 737–746. (10.1007/s10549-010-1261-9) 21113658 PMC3685185

[bib33] Clement JH Sanger J Hoffken K 1999 Expression of bone morphogenetic protein 6 in normal mammary tissue and breast cancer cell lines and its regulation by epidermal growth factor. International Journal of Cancer 80 250–256. (10.1002/(SICI)1097-0215(19990118)80:2<250::AID-IJC14>3.0.CO;2-D) 9935207

[bib31] Clement JH Marr N Meissner A Schwalbe M Sebald W Kliche KO Hoffken K Wolfl S 2000 Bone morphogenetic protein 2 (BMP-2) induces sequential changes of Id gene expression in the breast cancer cell line MCF-7. Journal of Cancer Research and Clinical Oncology 126 271–279. (10.1007/s004320050342) 10815762 PMC12165198

[bib32] Clement JH Raida M Sanger J Bicknell R Liu J Naumann A Geyer A Waldau A Hortschansky P Schmidt A 2005 Bone morphogenetic protein 2 (BMP-2) induces in vitro invasion and in vivo hormone independent growth of breast carcinoma cells. International Journal of Oncology 27 401–407. (10.3892/ijo.27.2.401) 16010421

[bib30] Clement F Xu X Donini CF Clement A Omarjee S Delay E Treilleux I Fervers B Le Romancer M Cohen PA 2017 Long-term exposure to bisphenol A or benzo(a)pyrene alters the fate of human mammary epithelial stem cells in response to BMP2 and BMP4, by pre-activating BMP signaling. Cell Death and Differentiation 24 155–166. (10.1038/cdd.2016.107) 27740625 PMC5260492

[bib34] Cochrane DR Bernales S Jacobsen BM Cittelly DM Howe EN D'Amato NC Spoelstra NS Edgerton SM Jean A Guerrero J 2014 Role of the androgen receptor in breast cancer and preclinical analysis of enzalutamide. Breast Cancer Research 16 R7 (10.1186/bcr3599) 24451109 PMC3978822

[bib35] Cowin P Wysolmerski J 2010 Molecular mechanisms guiding embryonic mammary gland development. Cold Spring Harbor Perspectives in Biology 2 a003251 (10.1101/cshperspect.a003251) 20484386 PMC2869520

[bib36] Cunha SI Pietras K 2011 ALK1 as an emerging target for antiangiogenic therapy of cancer. Blood 117 6999–7006. (10.1182/blood-2011-01-330142) 21467543 PMC3143549

[bib37] Dai J Kitagawa Y Zhang J Yao Z Mizokami A Cheng S Nor J McCauley LK Taichman RS Keller ET 2004 Vascular endothelial growth factor contributes to the prostate cancer-induced osteoblast differentiation mediated by bone morphogenetic protein. Cancer Research 64 994–999. (10.1158/0008-5472.CAN-03-1382) 14871830

[bib38] Davies SR Watkins G Douglas-Jones A Mansel RE Jiang WG 2008 Bone morphogenetic proteins 1 to 7 in human breast cancer, expression pattern and clinical/prognostic relevance. Journal of Experimental Therapeutics and Oncology 7 327–338. 19227012

[bib39] Davis H Raja E Miyazono K Tsubakihara Y Moustakas A 2016 Mechanisms of action of bone morphogenetic proteins in cancer. Cytokine and Growth Factor Reviews 27 81–92. (10.1016/j.cytogfr.2015.11.009) 26678814

[bib40] de Boeck M Cui C Mulder AA Jost CR Ikeno S Ten Dijke P 2016 Smad6 determines BMP-regulated invasive behaviour of breast cancer cells in a zebrafish xenograft model. Scientific Reports 6 24968 (10.1038/srep24968) 27113436 PMC4844967

[bib41] Deckers MM van Bezooijen RL van der Horst G Hoogendam J van Der Bent C Papapoulos SE Lowik CW 2002 Bone morphogenetic proteins stimulate angiogenesis through osteoblast-derived vascular endothelial growth factor A. Endocrinology 143 1545–1553. (10.1210/endo.143.4.8719) 11897714

[bib42] Derynck R Feng XH 1997 TGF-beta receptor signaling. Biochimica et Biophysica Acta 1333 F105–F150. 9395284 10.1016/s0304-419x(97)00017-6

[bib44] Du J Yang S Wang Z Zhai C Yuan W Lei R Zhang J Zhu T 2008 Bone morphogenetic protein 6 inhibit stress-induced breast cancer cells apoptosis via both Smad and p38 pathways. Journal of Cellular Biochemistry 103 1584–1597. (10.1002/jcb.21547) 17879955

[bib43] Du J Yang S An D Hu F Yuan W Zhai C Zhu T 2009 BMP-6 inhibits microRNA-21 expression in breast cancer through repressing deltaEF1 and AP-1. Cell Research 19 487–496. (10.1038/cr.2009.34) 19308091

[bib45] Dumont N Arteaga CL 2003 A kinase-inactive type II TGFbeta receptor impairs BMP signaling in human breast cancer cells. Biochemical and Biophysical Research Communications 301 108–112. (10.1016/S0006-291X(02)02977-7) 12535648

[bib46] Feng J Li L Zhang N Liu J Zhang L Gao H Wang G Li Y Zhang Y Li X 2017 Androgen and AR contribute to breast cancer development and metastasis: an insight of mechanisms. Oncogene 36 2775–2790. (10.1038/onc.2016.432) 27893717

[bib47] Ferlay J Soerjomataram I Dikshit R Eser S Mathers C Rebelo M Parkin DM Forman D Bray F 2015 Cancer incidence and mortality worldwide: sources, methods and major patterns in GLOBOCAN. 2012 International Journal of Cancer 136 E359–E386. (10.1002/ijc.29210) 25220842

[bib48] Forsman CL Ng BC Heinze RK Kuo C Sergi C Gopalakrishnan R Yee D Graf D Schwertfeger KL Petryk A 2013 BMP-binding protein twisted gastrulation is required in mammary gland epithelium for normal ductal elongation and myoepithelial compartmentalization. Developmental Biology 373 95–106. (10.1016/j.ydbio.2012.10.007) 23103586 PMC3508155

[bib49] Gao H Chakraborty G Lee-Lim AP Mo Q Decker M Vonica A Shen R Brogi E Brivanlou AH Giancotti FG 2012 The BMP inhibitor Coco reactivates breast cancer cells at lung metastatic sites. Cell 150 764–779. (10.1016/j.cell.2012.06.035) 22901808 PMC3711709

[bib50] Garulli C Kalogris C Pietrella L Bartolacci C Andreani C Falconi M Marchini C Amici A 2014 Dorsomorphin reverses the mesenchymal phenotype of breast cancer initiating cells by inhibition of bone morphogenetic protein signaling. Cellular Signalling 26 352–362. (10.1016/j.cellsig.2013.11.022) 24280125

[bib51] Gatza CE Elderbroom JL Oh SY Starr MD Nixon AB Blobe GC 2014 The balance of cell surface and soluble type III TGF-beta receptor regulates BMP signaling in normal and cancerous mammary epithelial cells. Neoplasia 16 489–500. (10.1016/j.neo.2014.05.008) 25077702 PMC4198744

[bib52] Gautschi O Tepper CG Purnell PR Izumiya Y Evans CP Green TP Desprez PY Lara PN Gandara DR Mack PC 2008 Regulation of Id1 expression by SRC: implications for targeting of the bone morphogenetic protein pathway in cancer. Cancer Research 68 2250–2258. (10.1158/0008-5472.CAN-07-6403) 18381431

[bib55] Ghosh Choudhury G Jin DC Kim Y Celeste A Ghosh-Choudhury N Abboud HE 1999 Bone morphogenetic protein-2 inhibits MAPK-dependent Elk-1 transactivation and DNA synthesis induced by EGF in mesangial cells. Biochemical and Biophysical Research Communications 258 490–496. (10.1006/bbrc.1999.0599) 10329414

[bib53] Ghosh-Choudhury N Ghosh-Choudhury G Celeste A Ghosh PM Moyer M Abboud SL Kreisberg J 2000a Bone morphogenetic protein-2 induces cyclin kinase inhibitor p21 and hypophosphorylation of retinoblastoma protein in estradiol-treated MCF-7 human breast cancer cells. Biochimica et Biophysica Acta 1497 186–196. (10.1016/S0167-4889(00)00060-4) 10903423

[bib54] Ghosh-Choudhury N Woodruff K Qi W Celeste A Abboud SL Ghosh Choudhury G 2000b Bone morphogenetic protein-2 blocks MDA MB 231 human breast cancer cell proliferation by inhibiting cyclin-dependent kinase-mediated retinoblastoma protein phosphorylation. Biochemical and Biophysical Research Communications 272 705–711. (10.1006/bbrc.2000.2844) 10860819

[bib56] Gul S Murad S Ehsan N Bloodsworth P Sultan A Faheem M 2015 Transcriptional up-regulation of BMP-4 and BMPR-II genes in the peripheral blood of breast cancer patients: a pilot study. Cancer Biomarkers 15 551–557. (10.3233/CBM-150494) 26406943 PMC12965442

[bib58] Guo X Wang XF 2009 Signaling cross-talk between TGF-beta/BMP and other pathways. Cell Research 19 71–88. (10.1038/cr.2008.302) 19002158 PMC3606489

[bib57] Guo D Huang J Gong J 2012 Bone morphogenetic protein 4 (BMP4) is required for migration and invasion of breast cancer. Molecular and Cellular Biochemistry 363 179–190. (10.1007/s11010-011-1170-1) 22167620

[bib59] Hanavadi S Martin TA Watkins G Mansel RE Jiang WG 2007 The role of growth differentiation factor-9 (GDF-9) and its analog, GDF-9b/BMP-15, in human breast cancer. Annals of Surgical Oncology 14 2159–2166. (10.1245/s10434-007-9397-5) 17453295

[bib60] Hawinkels LJ de Vinuesa AG Paauwe M Kruithof-de Julio M Wiercinska E Pardali E Mezzanotte L Keereweer S Braumuller TM Heijkants RC 2016 Activin receptor-like kinase 1 ligand trap reduces microvascular density and improves chemotherapy efficiency to various solid tumors. Clinical Cancer Research 22 96–106. (10.1158/1078-0432.CCR-15-0743) 26373572

[bib61] Helms MW Packeisen J August C Schittek B Boecker W Brandt BH Buerger H 2005 First evidence supporting a potential role for the BMP/SMAD pathway in the progression of oestrogen receptor-positive breast cancer. Journal of Pathology 206 366–376. (10.1002/path.1785) 15892165

[bib62] Hens JR Wysolmerski JJ 2005 Key stages of mammary gland development: molecular mechanisms involved in the formation of the embryonic mammary gland. Breast Cancer Research 7 220–224. (10.1186/bcr1306) 16168142 PMC1242158

[bib63] Hu F Zhang Y Li M Zhao L Chen J Yang S Zhang X 2016 BMP-6 inhibits the metastasis of MDA-MB-231 breast cancer cells by regulating MMP-1 expression. Oncology Reports 35 1823–1830. (10.3892/or.2015.4540) 26751737

[bib64] Ibrahim T Leong I Sanchez-Sweatman O Khokha R Sodek J Tenenbaum HC Ganss B Cheifetz S 2000 Expression of bone sialoprotein and osteopontin in breast cancer bone metastases. Clinical and Experimental Metastasis 18 253–260. 11315099 10.1023/a:1006754605901

[bib65] Imai Y Terai H Nomura-Furuwatari C Mizuno S Matsumoto K Nakamura T Takaoka K 2005 Hepatocyte growth factor contributes to fracture repair by upregulating the expression of BMP receptors. Journal of Bone and Mineral Research 20 1723–1730. (10.1359/JBMR.050607) 16160730

[bib66] Jin C Yang YA Anver MR Morris N Wang X Zhang YE 2009 Smad ubiquitination regulatory factor 2 promotes metastasis of breast cancer cells by enhancing migration and invasiveness. Cancer Research 69 735–740. (10.1158/0008-5472.CAN-08-1463) 19155312 PMC2639752

[bib67] Julien S Ivetic A Grigoriadis A QiZe D Burford B Sproviero D Picco G Gillett C Papp SL Schaffer L 2011 Selectin ligand sialyl-Lewis x antigen drives metastasis of hormone-dependent breast cancers. Cancer Research 71 7683–7693. (10.1158/0008-5472.CAN-11-1139) 22025563 PMC6485480

[bib68] Kapoor P Suva LJ Welch DR Donahue HJ 2008 Osteoprotegrin and the bone homing and colonization potential of breast cancer cells. Journal of Cellular Biochemistry 103 30–41. (10.1002/jcb.21382) 17471510

[bib69] Katsuno Y Hanyu A Kanda H Ishikawa Y Akiyama F Iwase T Ogata E Ehata S Miyazono K Imamura T 2008 Bone morphogenetic protein signaling enhances invasion and bone metastasis of breast cancer cells through Smad pathway. Oncogene 27 6322–6333. (10.1038/onc.2008.232) 18663362

[bib70] Ketolainen JM Alarmo EL Tuominen VJ Kallioniemi A 2010 Parallel inhibition of cell growth and induction of cell migration and invasion in breast cancer cells by bone morphogenetic protein 4. Breast Cancer Research and Treatment 124 377–386. (10.1007/s10549-010-0808-0) 20182795

[bib71] Konfortion J Jack RH Davies EA 2014 Coverage of common cancer types in UK national newspapers: a content analysis. BMJ Open 4 e004677 (10.1136/bmjopen-2013-004677) PMC4120380 25015469

[bib72] Kretzschmar M Doody J Massague J 1997 Opposing BMP and EGF signalling pathways converge on the TGF-beta family mediator Smad1. Nature 389 618–622. (10.1038/39348) 9335504

[bib73] Lamouille S Xu J Derynck R 2014 Molecular mechanisms of epithelial-mesenchymal transition. Nature Reviews Molecular Cell Biology 15 178–196. (10.1038/nrm3758) 24556840 PMC4240281

[bib74] Larue L Bellacosa A 2005 Epithelial-mesenchymal transition in development and cancer: role of phosphatidylinositol 3' kinase/AKT pathways. Oncogene 24 7443–7454. (10.1038/sj.onc.1209091) 16288291

[bib75] Laulan NB St-Pierre Y 2015 Bone morphogenetic protein 4 (BMP-4) and epidermal growth factor (EGF) inhibit metalloproteinase-9 (MMP-9) expression in cancer cells. Oncoscience 2 309–316. (10.18632/oncoscience.144) 25897433 PMC4394136

[bib76] Li J Ye L Sanders AJ Jiang WG 2012 Repulsive guidance molecule B (RGMB) plays negative roles in breast cancer by coordinating BMP signaling. Journal of Cellular Biochemistry 113 2523–2531. (10.1002/jcb.24128) 22415859

[bib77] Lian WJ Liu G Liu YJ Zhao ZW Yi T Zhou HY 2013 Downregulation of BMP6 enhances cell proliferation and chemoresistance via activation of the ERK signaling pathway in breast cancer. Oncology Reports 30 193–200. (10.3892/or.2013.2462) 23674072

[bib78] Lipton A Uzzo R Amato RJ Ellis GK Hakimian B Roodman GD Smith MR 2009 The science and practice of bone health in oncology: managing bone loss and metastasis in patients with solid tumors. Journal of the National Comprehensive Cancer Network 7 (Supplement 7) S1–S29. (10.6004/jnccn.2009.0080) PMC3047391 19878635

[bib79] Matsumoto Y Otsuka F Takano-Narazaki M Katsuyama T Nakamura E Tsukamoto N Inagaki K Sada KE Makino H 2013 Estrogen facilitates osteoblast differentiation by upregulating bone morphogenetic protein-4 signaling. Steroids 78 513–520. (10.1016/j.steroids.2013.02.011) 23499826

[bib80] Miyazono K 2008 Regulation of TGF-β family signaling by inhibitory smads. In The TGF-β Family. Eds Derynck R Miyazono K New York, NY, USA: Cold Spring Harbor Laboratory Press

[bib81] Mock K Preca BT Brummer T Brabletz S Stemmler MP Brabletz T 2015 The EMT-activator ZEB1 induces bone metastasis associated genes including BMP-inhibitors. Oncotarget 6 14399–14412. (10.18632/oncotarget.3882) 25973542 PMC4546475

[bib82] Montesano R 2007 Bone morphogenetic protein-4 abrogates lumen formation by mammary epithelial cells and promotes invasive growth. Biochemical and Biophysical Research Communications 353 817–822. (10.1016/j.bbrc.2006.12.109) 17189614

[bib83] Montesano R Sarkozi R Schramek H 2008 Bone morphogenetic protein-4 strongly potentiates growth factor-induced proliferation of mammary epithelial cells. Biochemical and Biophysical Research Communications 374 164–168. (10.1016/j.bbrc.2008.07.007) 18625198

[bib84] Moreau JE Anderson K Mauney JR Nguyen T Kaplan DL Rosenblatt M 2007 Tissue-engineered bone serves as a target for metastasis of human breast cancer in a mouse model. Cancer Research 67 10304–10308. (10.1158/0008-5472.CAN-07-2483) 17974972

[bib85] Nakajima Y Yamagishi T Hokari S Nakamura H 2000 Mechanisms involved in valvuloseptal endocardial cushion formation in early cardiogenesis: roles of transforming growth factor (TGF)-beta and bone morphogenetic protein (BMP). Anatomical Record 258 119–127. (10.1002/(SICI)1097-0185(20000201)258:2<119::AID-AR1>3.0.CO;2-U) 10645959

[bib86] Nohe A Keating E Knaus P Petersen NO 2004 Signal transduction of bone morphogenetic protein receptors. Cellular Signalling 16 291–299. (10.1016/j.cellsig.2003.08.011) 14687659

[bib87] Ong DB Colley SM Norman MR Kitazawa S Tobias JH 2004 Transcriptional regulation of a BMP-6 promoter by estrogen receptor alpha. Journal of Bone and Mineral Research 19 447–454. (10.1359/JBMR.0301249) 15040833

[bib88] Oskarsson T Batlle E Massague J 2014 Metastatic stem cells: sources, niches, and vital pathways. Cell Stem Cell 14 306–321. (10.1016/j.stem.2014.02.002) 24607405 PMC3998185

[bib89] Owens P Pickup MW Novitskiy SV Chytil A Gorska AE Aakre ME West J Moses HL 2012 Disruption of bone morphogenetic protein receptor 2 (BMPR2) in mammary tumors promotes metastases through cell autonomous and paracrine mediators. PNAS 109 2814–2819. (10.1073/pnas.1101139108) 21576484 PMC3286911

[bib91] Owens P Polikowsky H Pickup MW Gorska AE Jovanovic B Shaw AK Novitskiy SV Hong CC Moses HL 2013 Bone morphogenetic proteins stimulate mammary fibroblasts to promote mammary carcinoma cell invasion. PLoS ONE 8 e67533 (10.1371/journal.pone.0067533) 23840733 PMC3695869

[bib90] Owens P Pickup MW Novitskiy SV Giltnane JM Gorska AE Hopkins CR Hong CC Moses HL 2015 Inhibition of BMP signaling suppresses metastasis in mammary cancer. Oncogene 34 2437–2449. (10.1038/onc.2014.189) 24998846 PMC4689138

[bib92] Pal A Huang W Li X Toy KA Nikolovska-Coleska Z Kleer CG 2012 CCN6 modulates BMP signaling via the Smad-independent TAK1/p38 pathway, acting to suppress metastasis of breast cancer. Cancer Research 72 4818–4828. (10.1158/0008-5472.CAN-12-0154) 22805309 PMC3506182

[bib93] Perdigao-Henriques R Petrocca F Altschuler G Thomas MP Le MT Tan SM Hide W Lieberman J 2016 miR-200 promotes the mesenchymal to epithelial transition by suppressing multiple members of the Zeb2 and Snail1 transcriptional repressor complexes. Oncogene 35 158–172. (10.1038/onc.2015.69) 25798844

[bib94] Pickup MW Hover LD Guo Y Gorska AE Chytil A Novitskiy SV Moses HL Owens P 2015a Deletion of the BMP receptor BMPR1a impairs mammary tumor formation and metastasis. Oncotarget 6 22890–22904. (10.18632/oncotarget.4413) 26274893 PMC4673207

[bib95] Pickup MW Hover LD Polikowsky ER Chytil A Gorska AE Novitskiy SV Moses HL Owens P 2015b BMPR2 loss in fibroblasts promotes mammary carcinoma metastasis via increased inflammation. Molecular Oncology 9 179–191. (10.1016/j.molonc.2014.08.004) 25205038 PMC4277920

[bib96] Piek E Moustakas A Kurisaki A Heldin CH ten Dijke P 1999 TGF-(beta) type I receptor/ALK-5 and Smad proteins mediate epithelial to mesenchymal transdifferentiation in NMuMG breast epithelial cells. Journal of Cell Science 112 4557–4568. 10574705 10.1242/jcs.112.24.4557

[bib98] Pouliot F Labrie C 2002 Role of Smad1 and Smad4 proteins in the induction of p21WAF1,Cip1 during bone morphogenetic protein-induced growth arrest in human breast cancer cells. Journal of Endocrinology 172 187–198. (10.1677/joe.0.1720187) 11786386

[bib97] Pouliot F Blais A Labrie C 2003 Overexpression of a dominant negative type II bone morphogenetic protein receptor inhibits the growth of human breast cancer cells. Cancer Research 63 277–281. 12543773

[bib99] Rahman MS Akhtar N Jamil HM Banik RS Asaduzzaman SM 2015 TGF-beta/BMP signaling and other molecular events: regulation of osteoblastogenesis and bone formation. Bone Research 3 15005 (10.1038/boneres.2015.5) 26273537 PMC4472151

[bib100] Raida M Clement JH Ameri K Han C Leek RD Harris AL 2005a Expression of bone morphogenetic protein 2 in breast cancer cells inhibits hypoxic cell death. International Journal of Oncology 26 1465–1470. (10.3892/ijo.26.6.1465) 15870857

[bib101] Raida M Clement JH Leek RD Ameri K Bicknell R Niederwieser D Harris AL 2005b Bone morphogenetic protein 2 (BMP-2) and induction of tumor angiogenesis. Journal of Cancer Research and Clinical Oncology 131 741–750. (10.1007/s00432-005-0024-1) 16136355 PMC12161192

[bib102] Reinholz MM Iturria SJ Ingle JN Roche PC 2002 Differential gene expression of TGF-beta family members and osteopontin in breast tumor tissue: analysis by real-time quantitative PCR. Breast Cancer Research and Treatment 74 255–269. (10.1023/A:1016339120506) 12206515

[bib103] Ren W Liu Y Wan S Fei C Wang W Chen Y Zhang Z Wang T Wang J Zhou L 2014a BMP9 inhibits proliferation and metastasis of HER2-positive SK-BR-3 breast cancer cells through ERK1/2 and PI3K/AKT pathways. PLoS ONE 9 e96816 (10.1371/journal.pone.0096816) 24805814 PMC4013047

[bib104] Ren W Sun X Wang K Feng H Liu Y Fei C Wan S Wang W Luo J Shi Q 2014b BMP9 inhibits the bone metastasis of breast cancer cells by downregulating CCN2 (connective tissue growth factor, CTGF) expression. Molecular Biology Reports 41 1373–1383. (10.1007/s11033-013-2982-8) 24413988

[bib105] Ribatti D Nico B Ruggieri S Tamma R Simone G Mangia A 2016 Angiogenesis and antiangiogenesis in triple-negative breast cancer. Translational Oncology 9 453–457. (10.1016/j.tranon.2016.07.002) 27751350 PMC5067931

[bib106] Romano LA Runyan RB 2000 Slug is an essential target of TGFbeta2 signaling in the developing chicken heart. Developmental Biology 223 91–102. (10.1006/dbio.2000.9750) 10864463

[bib107] Royce ME Osman D 2015 Everolimus in the treatment of metastatic breast cancer. Breast Cancer 9 73–79. (10.4137/BCBCR.S29268) 26417203 PMC4571987

[bib108] Rucci N Teti A 2010 Osteomimicry: how tumor cells try to deceive the bone. Frontiers in Bioscience 2 907–915. (10.2741/s110) 20515833

[bib109] Sakai H Furihata M Matsuda C Takahashi M Miyazaki H Konakahara T Imamura T Okada T 2012 Augmented autocrine bone morphogenic protein (BMP) 7 signaling increases the metastatic potential of mouse breast cancer cells. Clinical and Experimental Metastasis 29 327–338. (10.1007/s10585-012-9453-9) 22274590

[bib110] Scherberich A Tucker RP Degen M Brown-Luedi M Andres AC Chiquet-Ehrismann R 2005 Tenascin-W is found in malignant mammary tumors, promotes alpha8 integrin-dependent motility and requires p38MAPK activity for BMP-2 and TNF-alpha induced expression in vitro. Oncogene 24 1525–1532. (10.1038/sj.onc.1208342) 15592496

[bib111] Schwalbe M Sanger J Eggers R Naumann A Schmidt A Hoffken K Clement JH 2003 Differential expression and regulation of bone morphogenetic protein 7 in breast cancer. International Journal of Oncology 23 89–95. (10.3892/ijo.23.1.89) 12792780

[bib112] Shon SK Kim A Kim JY Kim KI Yang Y Lim JS 2009 Bone morphogenetic protein-4 induced by NDRG2 expression inhibits MMP-9 activity in breast cancer cells. Biochemical and Biophysical Research Communications 385 198–203. (10.1016/j.bbrc.2009.05.038) 19450561

[bib114] Soda H Raymond E Sharma S Lawrence R Cerna C Gomez L Timony GA Von Hoff DD Izbicka E 1998 Antiproliferative effects of recombinant human bone morphogenetic protein-2 on human tumor colony-forming units. Anticancer Drugs 9 327–331. (10.1097/00001813-199804000-00006) 9635923

[bib115] Steger GG Bartsch R 2011 Denosumab for the treatment of bone metastases in breast cancer: evidence and opinion. Therapeutic Advances in Medical Oncology 3 233–243. (10.1177/1758834011412656) 21957430 PMC3169928

[bib116] Steinert S Kroll TC Taubert I Pusch L Hortschansky P Hoffken K Wolfl S Clement JH 2008 Differential expression of cancer-related genes by single and permanent exposure to bone morphogenetic protein 2. Journal of Cancer Research and Clinical Oncology 134 1237–1245. (10.1007/s00432-008-0396-0) 18446370 PMC12161735

[bib117] Suvannasankha A Chirgwin JM 2014 Role of bone-anabolic agents in the treatment of breast cancer bone metastases. Breast Cancer Research 16 484 (10.1186/s13058-014-0484-9) 25757219 PMC4429670

[bib118] Takahashi M Otsuka F Miyoshi T Otani H Goto J Yamashita M Ogura T Makino H Doihara H 2008 Bone morphogenetic protein 6 (BMP6) and BMP7 inhibit estrogen-induced proliferation of breast cancer cells by suppressing p38 mitogen-activated protein kinase activation. Journal of Endocrinology 199 445–455. (10.1677/JOE-08-0226) 18780779

[bib119] Tan CC Li GX Tan LD Du X Li XQ He R Wang QS Feng YM 2016 Breast cancer cells obtain an osteomimetic feature via epithelial-mesenchymal transition that have undergone BMP2/RUNX2 signaling pathway induction. Oncotarget 7 79688–79705. (10.18632/oncotarget.12939) 27806311 PMC5346745

[bib120] Tarragona M Pavlovic M Arnal-Estape A Urosevic J Morales M Guiu M Planet E Gonzalez-Suarez E Gomis RR 2012 Identification of NOG as a specific breast cancer bone metastasis-supporting gene. Journal of Biological Chemistry 287 21346–21355. (10.1074/jbc.M112.355834) 22547073 PMC3375555

[bib121] van den Wijngaard A Mulder WR Dijkema R Boersma CJ Mosselman S van Zoelen EJ Olijve W 2000 Antiestrogens specifically up-regulate bone morphogenetic protein-4 promoter activity in human osteoblastic cells. Molecular Endocrinology 14 623–633. (10.1210/mend.14.5.0463) 10809227

[bib122] Varga AC Wrana JL 2005 The disparate role of BMP in stem cell biology. Oncogene 24 5713–5721. (10.1038/sj.onc.1208919) 16123804

[bib123] Waite KA Eng C 2003 BMP2 exposure results in decreased PTEN protein degradation and increased PTEN levels. Human Molecular Genetics 12 679–684. (10.1093/hmg/ddg069) 12620973

[bib124] Walsh DW Godson C Brazil DP Martin F 2010 Extracellular BMP-antagonist regulation in development and disease: tied up in knots. Trends in Cell Biology 20 244–256. (10.1016/j.tcb.2010.01.008) 20188563

[bib126] Wang HC 2015 The distribution and expression of BAMBI in breast cancer cell lines. Open Access Library Journal 2 8.

[bib125] Wang D Huang P Zhu B Sun L Huang Q Wang J 2012 Induction of estrogen receptor alpha-36 expression by bone morphogenetic protein 2 in breast cancer cell lines. Molecular Medicine Reports 6 591–596. (10.3892/mmr.2012.945) 22711074

[bib127] Wang K Feng H Ren W Sun X Luo J Tang M Zhou L Weng Y He TC Zhang Y 2011 BMP9 inhibits the proliferation and invasiveness of breast cancer cells MDA-MB-231. Journal of Cancer Research and Clinical Oncology 137 1687–1696. (10.1007/s00432-011-1047-4) 21892652 PMC11827914

[bib128] Woodward WA Chen MS Behbod F Rosen JM 2005 On mammary stem cells. Journal of Cell Science 118 3585–3594. (10.1242/jcs.02532) 16105882

[bib129] Wu L Wu Y Gathings B Wan M Li X Grizzle W Liu Z Lu C Mao Z Cao X 2003 Smad4 as a transcription corepressor for estrogen receptor alpha. Journal of Biological Chemistry 278 15192–15200. (10.1074/jbc.M212332200) 12576474

[bib130] Xie Y Avello M Schirle M McWhinnie E Feng Y Bric-Furlong E Wilson C Nathans R Zhang J Kirschner MW 2013 Deubiquitinase FAM/USP9X interacts with the E3 ubiquitin ligase SMURF1 protein and protects it from ligase activity-dependent self-degradation. Journal of Biological Chemistry 288 2976–2985. (10.1074/jbc.M112.430066) 23184937 PMC3561522

[bib131] Yamamoto T Saatcioglu F Matsuda T 2002 Cross-talk between bone morphogenic proteins and estrogen receptor signaling. Endocrinology 143 2635–2642. (10.1210/endo.143.7.8877) 12072396

[bib132] Yamashita H Ogiya A Shien T Horimoto Y Masuda N Inao T Osako T Takahashi M Endo Y Hosoda M 2016 Clinicopathological factors predicting early and late distant recurrence in estrogen receptor-positive, HER2-negative breast cancer. Breast Cancer 23 830–843. (10.1007/s12282-015-0649-0) 26467036

[bib133] Yan H Zhu S Song C Liu N Kang J 2012 Bone morphogenetic protein (BMP) signaling regulates mitotic checkpoint protein levels in human breast cancer cells. Cellular Signalling 24 961–968. (10.1016/j.cellsig.2011.12.019) 22234345

[bib135] Yang S Du J Wang Z Yuan W Qiao Y Zhang M Zhang J Gao S Yin J Sun B 2007 BMP-6 promotes E-cadherin expression through repressing deltaEF1 in breast cancer cells. BMC Cancer 7 211 (10.1186/1471-2407-7-211) 17997862 PMC2217560

[bib134] Yang S Du J Wang Z Yan J Yuan W Zhang J Zhu T 2009 Dual mechanism of deltaEF1 expression regulated by bone morphogenetic protein-6 in breast cancer. International Journal of Biochemistry and Cell Biology 41 853–861. (10.1016/j.biocel.2008.08.030) 18805502

[bib136] Yardley DA 2016 Pharmacologic management of bone-related complications and bone metastases in postmenopausal women with hormone receptor-positive breast cancer. Breast Cancer 8 73–82. (10.2147/BCTT.S97963) 27217795 PMC4861000

[bib139] Ye L Jiang WG 2016 Bone morphogenetic proteins in tumour associated angiogenesis and implication in cancer therapies. Cancer Letters 380 586–597. (10.1016/j.canlet.2015.10.036) 26639195

[bib140] Ye L Lewis-Russell JM Davies G Sanders AJ Kynaston H Jiang WG 2007a Hepatocyte growth factor up-regulates the expression of the bone morphogenetic protein (BMP) receptors, BMPR-IB and BMPR-II, in human prostate cancer cells. International Journal of Oncology 30 521–529. (10.3892/ijo.30.2.521) 17203235

[bib141] Ye L Lewis-Russell JM Kyanaston HG Jiang WG 2007b Bone morphogenetic proteins and their receptor signaling in prostate cancer. Histology and Histopathology 22 1129–1147. (10.14670/HH-22.1129) 17616940

[bib142] Ye L Lewis-Russell JM Sanders AJ Kynaston H Jiang WG 2008 HGF/SF up-regulates the expression of bone morphogenetic protein 7 in prostate cancer cells. Urologic Oncology 26 190–197. (10.1016/j.urolonc.2007.03.027) 18312940

[bib138] Ye L Bokobza SM Jiang WG 2009 Bone morphogenetic proteins in development and progression of breast cancer and therapeutic potential. International Journal of Molecular Medicine 24 591–597. (10.3892/ijmm_00000269) 19787192

[bib137] Ye L Bokobza S Li J Moazzam M Chen J Mansel RE Jiang WG 2010 Bone morphogenetic protein-10 (BMP-10) inhibits aggressiveness of breast cancer cells and correlates with poor prognosis in breast cancer. Cancer Science 101 2137–2144. (10.1111/­j.1349-7006.2010.01648.x) 20608934 PMC11158251

[bib143] Yeh LC Lee JC 1999 Osteogenic protein-1 increases gene expression of vascular endothelial growth factor in primary cultures of fetal rat calvaria cells. Molecular and Cellular Endocrinology 153 113–124. (10.1016/S0303-7207(99)00076-3) 10459859

[bib146] Zhang M Yan JD Zhang L Wang Q Lu SJ Zhang J Zhu TH 2005 Activation of bone morphogenetic protein-6 gene transcription in MCF-7 cells by estrogen. Chinese Medical Journal 118 1629–1636. 16232348

[bib145] Zhang M Wang Q Yuan W Yang S Wang X Yan JD Du J Yin J Gao SY Sun BC 2007 Epigenetic regulation of bone morphogenetic protein-6 gene expression in breast cancer cells. Journal of Steroid Biochemistry and Molecular Biology 105 91–97. (10.1016/j.jsbmb.2007.01.002) 17574840

[bib148] Zhang XH Giuliano M Trivedi MV Schiff R Osborne CK 2013 Metastasis dormancy in estrogen receptor-positive breast cancer. Clinical Cancer Research 19 6389-6397. (10.1158/1078-0432.CCR-13-0838) 24298069 PMC3878717

[bib147] Zhang Q Liang F Ke Y Huo Y Li M Li Y Yue J 2015 Overexpression of neogenin inhibits cell proliferation and induces apoptosis in human MDA-MB-231 breast carcinoma cells. Oncology Reports. (10.3892/or.2015.4004) 25998984

[bib144] Zhang L Ye Y Long X Xiao P Ren X Yu J 2016 BMP signaling and its paradoxical effects in tumorigenesis and dissemination. Oncotarget 7 78206–78218. (10.18632/oncotarget.12151) 27661009 PMC5363655

